# The Effects of Preservatives on Antibiotic- and Preservative-Resistant Microbes and Nitrogen/Sulfur Cycle Associated Microbial Communities in Freshwater River Sediments

**DOI:** 10.3390/antibiotics12071082

**Published:** 2023-06-21

**Authors:** Chien-Sen Liao, Xuan-Di Cao, Wei-Chen Lee, Chu-Wen Yang

**Affiliations:** 1 Department of Biological Science and Technology, I-Shou University, Kaohsiung 82445, Taiwan; 2 Institute of Biotechnology and Chemical Engineering, I-Shou University, Kaohsiung 84001, Taiwan; 3 Department of Microbiology, Soochow University, Taipei City 111002, Taiwan

**Keywords:** preservative-resistant microbes, nitrogen cycle, sulfur cycle, antibiotic-resistant microbes, freshwater river sediments

## Abstract

The intensive use of benzoic acid (BA), 4-hydroxybenzoic acid (HB), and dehydroacetate (DHA) as additives and preservatives in cosmetics and foods causes emerging environmental pollutions. Anthropogenic releases of BA, HB and DHA are primarily emissions into water and soil. However, few studies investigate the effects of BA, HB and DHA on microbial communities in freshwater river sediments. The aim of this study is to reveal the effects of BA, HB and DHA on microbial communities in freshwater river sediments. Tetracycline-, sulfamethoxazole- and preservative-resistant microbes were increased in the river sediments treated with BA, HB and DHA. The relative abundances of methanogen- and xenobiotic-degradation-associated microbial communities were also increased in the BA-, HB- and DHA-treated sediments. The relative abundance of four nitrogen cycle associated microbial groups (anammox, nitrogen fixation, denitrification, and dissimilatory nitrate reduction) were increased after the eighth week in the BA-, HB- and DHA-treated sediments. For the sulfur cycle, the relative abundance of thiosulfate oxidation associated microbial communities were increased after the eighth week in the BA-, HB- and DHA-treated sediments. Results of this study provide insight into the effects of BA, HB and DHA on antibiotic resistance, nitrogen cycle, sulfur cycle, drug resistance and methane production in freshwater aquatic environments.

## 1. Introduction

Benzoic acid (BA) exhibits antimicrobial activity around pH 2.5–4.5. It is usually used as a preservative in acid foodstuffs [1]. BA can be used in a great variety of foods, including egg products, soft drinks, seafoods, meats, sauces, juices, canned foods, beverages, condiments, fruits and vegetable products [2]. Mixtures of benzoic acid with other acids (such as sorbic, propionic, citric, lactic, ascorbic acids, nitrates and nitrites) can be used in fermented vegetables [3]. BA has a wide range of antimicrobial activities on microorganisms involved in food spoilage and poisoning. BA is effective against yeasts and molds (including *Aspergillus*, *Candida*, *Kloeckera*, *Eurotium*, *Debaryomyces*, *Saccharomyces*, *Penicillium*, *Pichia*, *Kluyveromyces* and *Zygosaccharomyces*) [4,5]. BA is also effective against many bacteria (including *Escherichia coli*, *Listeria monocytogenes*, *Lactobacillus brevis*, *Staphylococcus aureus*, *Lactobacillus plantarum*, *Pseudomonas aeruginosa*, *Leuconostoc dextranicum* and *Leuconostoc mesenteroides*) [1]. BA can reduce the bacteria to fungi ratio in the soil, and lead to changes in soil microbial communities associated with soilborne peanut diseases [6].

Currently, 4-hydroxybenzoic acid (HB) has emerged as a promising precursor/intermediate for several bioproducts, with potential applications for food, cosmetics, pharmacy, fungicides, etc. [6]. HB can be used as a preservative in the cosmetics, pharmaceutical and food industries [7]. HB can be used to synthesize parabens, such as ethyl 4-hydroxybenzoate and methyl 4-hydroxybenzoate, which serve as preservatives in cosmetics, pharmaceuticals, food and beverages [8]. HB is also a key component in the production of high-performance liquid crystal polymers, with increasing applications in the thermoplastic industry [9]. In addition, HB exhibits various biological properties, including hypoglycemic, anti-inflammatory, antiviral and antioxidative activities [10].

Dehydroacetate (DHA) is usually added to pharmaceutical drugs, foods, beverages, and animal feed due to its broad-spectrum antibacterial and antifungal activities [11,12,13,14]. Due to its inhibitory effects on *Penicillium digitatum* and *Penicillium italicum*, DHA is an alternative fungicide for the control of green and blue molds in citrus fruit [15]. DHA has adverse effects on vertebrates. It has been reported that sodium dehydroacetate induces Ca^2+^ imbalance associated cardiovascular toxicity in zebrafishes [16]. Sodium dehydroacetate exposure decreases hypoxia tolerance and locomotor persistence in zebrafishes [17].

Occurrences of BA, HB and DHA in the environment are found as a consequence of their widespread production and utilization. Anthropogenic releases of BA, HB and DHA are primarily emissions into water and soil [1]. HB is not only a precursor, but also a metabolite of parabens, which have been detected in WWTP effluents. Residual amounts of parabens and their metabolites were released into the surface water of freshwater rivers and other aquatic environments [18]. Parabens and HB have become chemicals of emerging concern for environments and humans [19]. DHA is used worldwide up to 0.6% in cosmetics and personal care products [20]. The heavy use of DHA inevitably causes environmental pollution. However, few studies investigate the fates and distributions of DHA in ecosystems.

Streams and rivers are important freshwater ecosystems with great significance to human life. The global nitrogen cycle has been largely affected by human activities, especially in rivers and streams, which led to hypoxic zone formation, eutrophication and increased N_2_O (a greenhouse gas) production [21]. In most streams and rivers, water above the sediment surface is under oxic conditions. An oxygen gradient is present at the sediment–water interface. Nitrification occurs in the oxic upper layer of sediment. The anaerobic denitrification, anammox, and dissimilatory nitrate reduction pathways occur in the anoxic subsurface sediment [22]. Moreover, it was found that the nitrogen cycle can be coupled with the sulfur cycle by a microbe-mediated process (sulfammox) in aquatic ecosystems [23]. Many studies reported that many antibiotics have significant side effects on the nitrogen cycle [24]. For instance, fluoroquinolones and sulfonamides were found to inhibit denitrification [25]. Moreover, the anammox process was found to be inhibited by oxytetracycline, tetracycline hydrochloride, sulfathiazole and chloramphenicol [26]. The environmental impacts of preservatives, such as BA, HB and DHA, have raised increasing concerns. However, little is known about the effects of BA, HB and DHA on microbial communities in freshwater river sediments. The aim of this study is to reveal the effects of BA, HB and DHA on antimicrobial-resistant, preservative-resistant, nitrogen/sulfur cycle and xenobiotic-degrading microbial communities in freshwater river sediments.

## 2. Results

### 2.1. Increase in the Tetracycline-, Sulfamethoxazole- and Preservative-Resistant Microbes in Sediments

**Figure 1:**
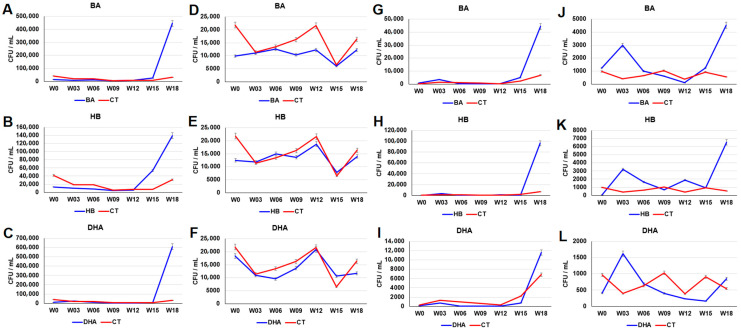
The plate counts of the sulfamethoxazole- (**A**–**F**) and tetracycline- (**G**–**L**) resistant microbes in the BA-, HB- and DHA-treated river sediments. (**A**–**C**, **G**–**I**): the aerobic culture. (**D**–**F**, **J**–**L**): the anaerobic culture. The *Y*-axis indicates the colony forming unit per mL (CFU/mL). The *X*-axis indicates weeks (0–18th week). Data from triplicate assays are presented as the mean ± SD. CT: control; BA: benzoic acid; HB: 4-hydroxybenzoate; DHA: dehydroacetic acid.

Plate counting was used to examine the number of bacteria in preservative-treated sediments. As shown in Figure S2, the total plate counts of the aerobic cultures of the BA-, HB- and DHA-treated sediments was less than the plate counts of aerobic cultures of the control sediments. The order of growth inhibition ability after the 15th week for the aerobic microbes in sediments is DHA > HB > BA (Figure S1A–C). The order of growth inhibition ability after the 15th week for the anaerobic microbes in sediments is BA > HB ≅ DHA (Figure S1D–F). The plate counts of the sulfamethoxazole-resistant microbes in the aerobic cultures of the BA-, HB- and DHA-treated sediments on the 18th week were (BA: 4.46 × 10^5^ ± 2.23 × 10^4^ CFU/mL vs. CT: 3.09 × 10^4^ ± 1.54 × 10^3^ CFU/mL, *p* value of *t* test = 5.57 × 10^−6^), (HB: 1.39 × 10^5^ ± 6.98 × 10^3^ CFU/mL vs. CT: 3.09 × 10^4^ ± 1.54 × 10^3^ CFU/mL, *p* value of *t* test = 1.23 × 10^−5^), and (DHA: 6.10 × 10^5^ ± 3.05 × 10^4^ CFU/mL vs. CT: 3.09 × 10^4^ ± 1.54 × 10^3^ CFU/mL, *p* value of *t* test = 5.12 × 10^−6^), respectively (Figure 1A–C). In contrast, the plate counts of the sulfamethoxazole-resistant microbes in the anaerobic cultures of the HB- and DHA-treated sediments showed a similar profile to the control sediments (Figure 1E,F). The plate counts of the tetracycline-resistant microbes in the aerobic cultures of the BA-, HB- and DHA-treated sediments on the 18th week were (BA: 4.42 × 10^4^ ± 2.21 × 10^3^ CFU/mL vs. CT: 6.83 × 10^3^ ± 3.41 × 10^2^ CFU/mL, *p* value of *t* test = 8.47 × 10^−6^), (HB: 9.62 × 10^4^ ± 4.81 × 10^3^ CFU/mL vs. CT: 6.83 × 10^3^ ± 3.41 × 10^2^ CFU/mL, *p* value of *t* test = 5.61 × 10^−6^), and (DHA: 1.16 × 10^4^ ± 5.81 × 10^2^ CFU/mL vs. CT: 6.83 × 10^3^ ± 3.41 × 10^2^ CFU/mL, *p* value of *t* test = 2.49 × 10^−4^), respectively (Figure 1G–I). The plate counts of the tetracycline-resistant microbes in the anaerobic cultures of the BA- and HB-treated sediments on the 18th week were (BA: 4.53 × 10^3^ ± 2.26 × 10^2^ CFU/mL vs. CT: 5.46 × 10^2^ ± 2.70 × 10^1^ CFU/mL, *p* value of *t* test = 7.12 × 10^−6^) and (HB: 6.54 × 10^3^ ± 2.21 × 10^3^ CFU/mL vs. CT: 5.46 × 10^2^ ± 2.70 × 10^1^ CFU/mL, *p* value of *t* test = 5.95 × 10^−6^), respectively (Figure 1J–K). The plate counts of the tetracycline-resistant microbes in the anaerobic cultures of the DHA-treated sediment were not significantly increased (Figure 1L).

**Figure 2:**
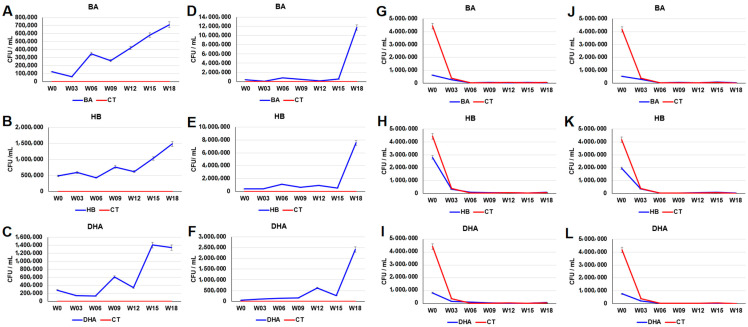
The plate counts of the BA-, HB- and DHA- (**A**–**F**) and penicillin- (**G**–**L**) resistant microbes in the BA-, HB- and DHA-treated river sediments. (**A**–**C**,**G**–**I**): the aerobic culture. (**D**–**F**,**J**–**L**): the anaerobic culture. The *Y*-axis indicates the colony forming unit per mL (CFU/mL). The *X*-axis indicates weeks (0–18th week). BA, HB and DHA indicate both treated and resistant preservatives. Data from triplicate assays are presented as the mean ± SD. CT: control; BA: benzoic acid; HB: 4-hydroxybenzoate; DHA: dehydroacetic acid.

The plate counts of the BA-, HB- and DHA-resistant microbes in the aerobic cultures of the BA-, HB- and DHA-treated sediments on the 18th week were (BA: 7.10 × 10^5^ ± 3.55 × 10^4^ CFU/mL vs. CT: 5.60 × 10^2^ ± 2.80 × 10^1^ CFU/mL, *p* value of *t* test = 4.16 × 10^−6^), (HB: 1.48 × 10^6^ ± 7.40 × 10^4^ CFU/mL vs. CT: 5.60 × 10^2^ ± 2.80 × 10^1^ CFU/mL, *p* value of *t* test = 4.15 × 10^−6^), and (DHA: 1.34 × 10^6^ ± 6.71 × 10^4^ CFU/mL vs. CT: 5.60 × 10^2^ ± 2.80 × 10^1^ CFU/mL, *p* value of *t* test = 4.15 × 10^−6^), respectively (Figure 2A–C). The plate counts of BA-, HB- and DHA-resistant microbes in the anaerobic cultures of the BA-, HB- and DHA-treated sediments on the 18th week were (BA: 1.17 × 10^7^ ± 5.88 × 10^5^ CFU/mL vs. CT: 3.80 × 10^2^ ± 1.90 × 10^1^ CFU/mL, *p* value of *t* test = 4.14 × 10^−6^), (HB: 7.54 × 10^6^ ± 5.88 × 10^5^ CFU/mL vs. CT: 3.80 × 10^2^ ± 1.90 × 10^1^ CFU/mL, *p* value of *t* test = 4.14 × 10^−6^), and (DHA: 2.42 × 10^6^ ± 1.21 × 10^5^ CFU/mL vs. CT: 3.80 × 10^2^ ± 1.90 × 10^1^ CFU/mL, *p* value of *t* test = 4.15 × 10^−6^), respectively (Figure 2D–F). The plate counts of penicillin-resistant microbes in both aerobic and anaerobic cultures of the BA-, HB- and DHA-treated sediments drastically decreased before the third week (Figure 2G–L). The plate counts of penicillin-resistant microbes in aerobic cultures of the BA-, HB- and DHA-treated sediments before the third week were (BA: 6.23 × 10^5^ ± 3.12 × 10^4^ CFU/mL vs. CT: 4.44 × 10^6^ ± 2.22 × 10^5^ CFU/mL, *p* value of *t* test = 7.87 × 10^−6^), (HB: 2.79 × 10^6^ ± 1.40 × 10^5^ CFU/mL vs. CT: 4.44 × 10^6^ ± 2.22 × 10^5^ CFU/mL, *p* value of *t* test = 3.66 × 10^−4^), and (DHA: 8.23 × 10^5^ ± 4.12 × 10^4^ CFU/mL vs. CT: 4.44 × 10^6^ ± 2.22 × 10^5^ CFU/mL, *p* value of *t* test = 1.00 × 10^−5^), respectively (Figure 2G–I). The plate counts of penicillin-resistant microbes in anaerobic cultures of the BA-, HB- and DHA-treated sediments the third week were (BA: 5.13 × 10^5^ ± 2.57 × 10^4^ CFU/mL vs. CT: 4.17 × 10^6^ ± 2.09 × 10^5^ CFU/mL, *p* value of *t* test = 7.21 × 10^−6^), (HB: 1.95 × 10^6^ ± 9.75 × 10^4^ CFU/mL vs. CT: 4.17 × 10^6^ ± 2.09 × 10^5^ CFU/mL, *p* value of *t* test = 7.50 × 10^−5^), and (DHA: 7.47 × 10^5^ ± 3.73 × 10^4^ CFU/mL vs. CT: 4.17 × 10^6^ ± 2.09 × 10^5^ CFU/mL, *p* value of *t* test = 9.68 × 10^−6^), respectively (Figure 2J–L).

### 2.2. Analysis of Chemical Compositions and Oxidation-Reduction Potential (ORP) in Waters

The continuous addition of 20 ppm BA, HB or DHA every week did not result in accumulation of high levels of preservatives in the river waters of the fish tanks (Figure S2). DHA exhibited the lowest level among the three preservatives. The residual BA and HB exhibiting very similar profiles may be due to similar chemical structures and degradation pathways of the two preservatives.

**Figure 3:**
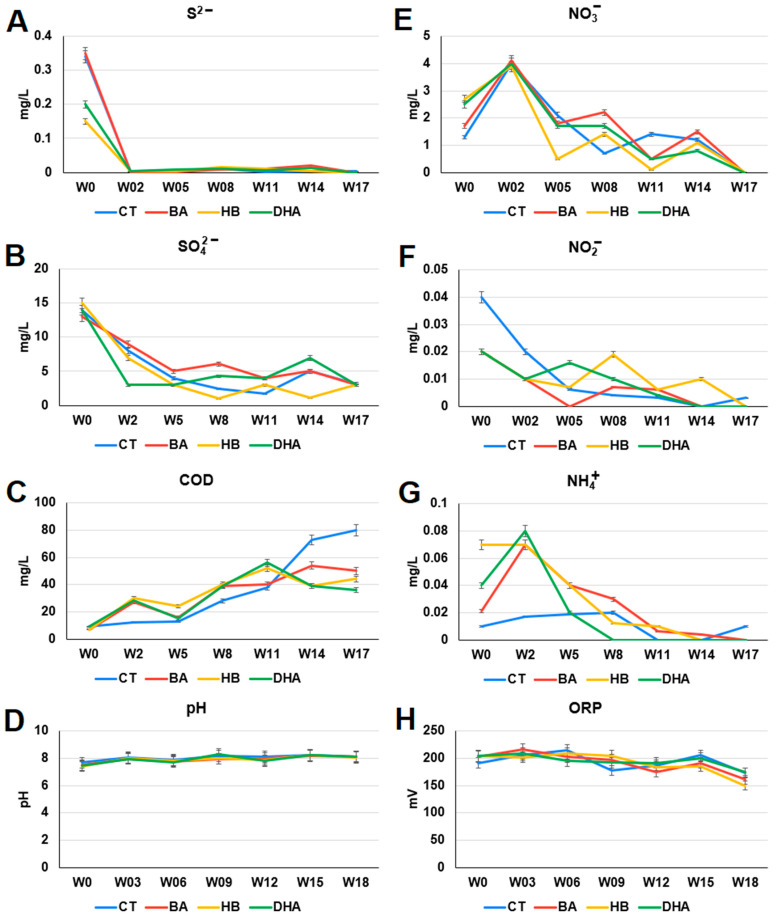
The chemical compositions of the river waters. (**A**) sulfide (S^2−^), (**B**) sulfate (SO_4_^2−^), (**C**) chemical oxygen demand (COD), (**D**) pH, (**E**) nitrate (NO_3_^−^), (**F**) nitrite (NO_2_^−^), (**G**) ammonium (NH_4_^+^), and (**H**) oxidation-reduction potential (ORP). The *X*-axis indicates weeks (0–18th week). Data from triplicate assays are presented as the mean ± SD. CT: control; BA: benzoic acid; HB: 4-hydroxybenzoate; DHA: dehydroacetic acid.

The chemical compositions and the oxidation-reduction potential (ORP) in control and preservative-treated river water were analyzed (Figure 3). The sulfide (S^2−^) levels of the HB- and DHA-treated river waters (HB: 1.50 × 10^−1^ ± 7.50 × 10^−3^ mg/L, DHA: 2.00 × 10^−1^ ± 1.00 × 10^−2^ mg/L) were lower than that of the control river water (CT: 3.40 × 10^−1^ ± 1.75 × 10^−2^ mg/L) on the 0th week (*p*-values of *t* test: HB: 5.97 × 10^−5^, DHA: 2.51 × 10^−4^) (Figure 3A). The COD levels of the preservative-treated river water (BA: 5.05 × 10^1^ ± 2.52 × 10^0^ mg/L, HB: 4.43 × 10^1^ ± 2.21 × 10^0^ mg/L, DHA: 3.66 × 10^1^ ± 1.83 × 10^0^ mg/L) were lower than that of the control river water (CT: 8.01 × 10^1^ ± 4.00 × 10^0^ mg/L) on the 17th week (*p*-values of *t* test: BA: 3.96 × 10^4^, HB: 1.68 × 10^4^, DHA: 6.62 × 10^5^) (Figure 3C). The nitrite (NO_2_^−^) levels of the preservative-treated river water (BA: 2.00 × 10^−2^ ± 1.00 × 10^−3^ mg/L, HB: 2.00 × 10^−2^ ± 1.00 × 10^−3^ mg/L, DHA: 2.00 × 10^−2^ ± 1.00 × 10^−3^ mg/L) were lower than that of the control river water (CT: 4.00 × 10^−2^ ± 2.00 × 10^−3^ mg/L) on the 0th week (*p*-values of *t* test: BA: 1.01 × 10^−4^, HB: 1.01 × 10^−4^, DHA: 1.01 × 10^−4^) (Figure 3F). The ammonium (NH_4_^+^) levels of the preservative-treated river waters (BA: 7.00 × 10^−2^ ± 3.50 × 10^−3^ mg/L, HB: 7.00 × 10^−2^ ± 3.50 × 10^−3^ mg/L, DHA: 8.00 × 10^−2^ ± 4.00 × 10^−3^ mg/L) were higher than that of the control river water (CT: 1.70 × 10^−2^ ± 8.50 × 10^−4^ mg/L) on the second week (*p*-values of *t* test: BA: 1.41 × 10^−5^, HB: 1.41 × 10^−5^, DHA: 1.17 × 10^−5^) (Figure 3G). The profiles of, pH and ORP were similar between the control and the preservative-treated river waters (Figure 3D,H).

### 2.3. Analysis of Microbial Community Compositions

**Figure 4:**
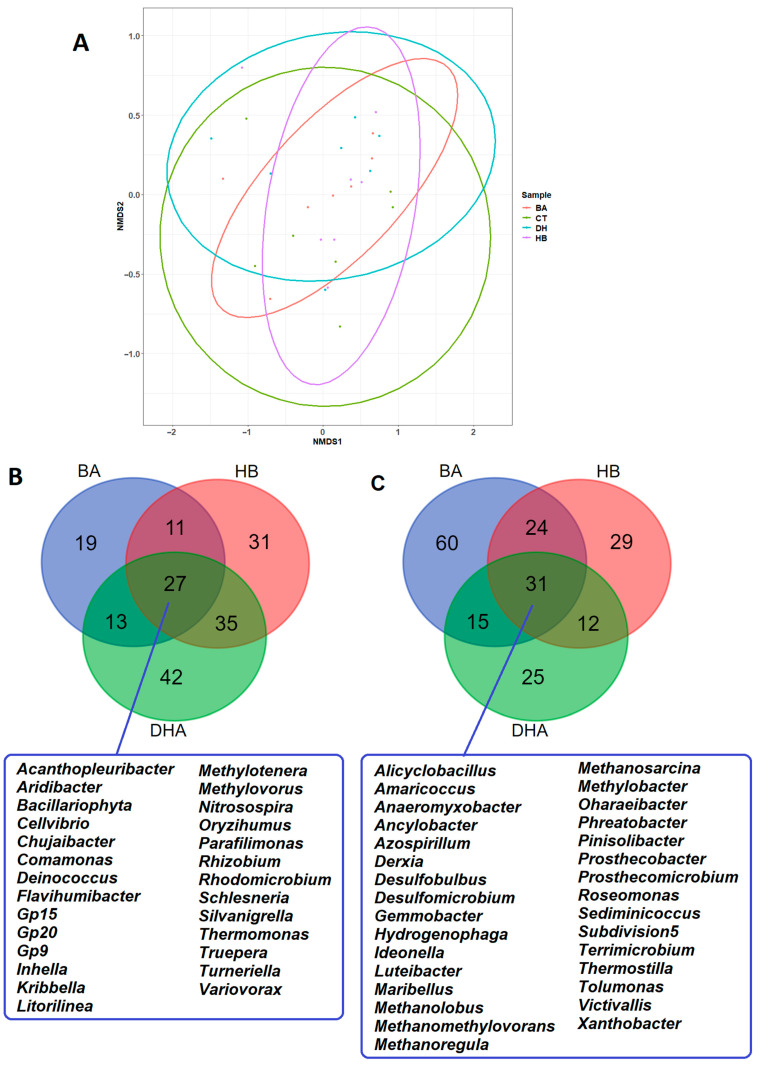
Identification of common and different microbial genera among BA-, HB- and DHA-treated river sediments. (**A**) Comparison (NMDS analysis) of microbiomes among BA-, HB- and DHA-treated river sediments. (**B**) Venn diagram analysis and number of microbial genera decreased in BA-, HB- and DHA-treated river sediments. (**C**) Venn diagram analysis and number of microbial genera increased in BA-, HB- and DHA-treated river sediments. CT: control; BA: benzoic acid; HB: 4-hydroxybenzoate; DHA: dehydroacetic acid.

The next generation sequencing of the 16S rRNA gene was used to analyze the microbial community compositions in river sediments. Alpha diversities (Shannon, Simpson, Chao 1 and ACE) of microbial community compositions in river sediments are shown in Figure S3. There is no significant difference among alpha diversities (Kruskal–Wallis tests, *p* values > 0.05). Overlapping ellipses in the result of the NMDS analysis indicates the presence of a core microbial community in sediments (Figure 4A). The *p* and R values of ANOSIM analysis are 0.5264 and −0.01296, respectively. There is no statistical significance in beta diversity. The relative abundance of twenty-seven microbial genera were decreased in the preservative-treated river sediments (Figure 4B). The relative abundance of thirty-one microbial genera (including four methanogens: *Methanolobus*, *Methanomethylovorans*, *Methanoregula*, *Methanosarcina*) were increased in the preservative-treated river sediments (Figure 4C). The details of relative abundances of these microbes are shown in Tables S1 and S2.

### 2.4. Microbial Community Associated with Nitrogen Cycle

**Figure 5:**
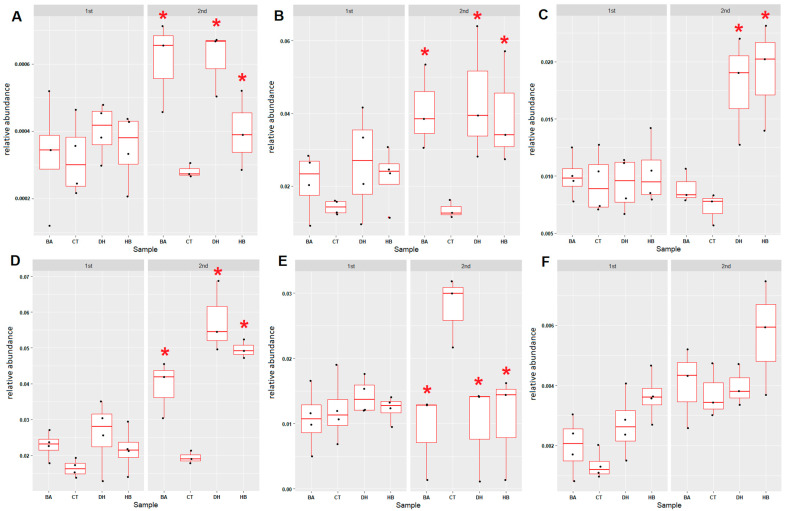
Overall relative abundance changes in nitrogen-cycle-associated microbial communities in the BA-, HB- and DHA-treated river sediments. (**A**) Anammox (anaerobic ammonium oxidation). (**B**) Nitrogen fixation (M00175: nitrogen => ammonia). (**C**) Denitrification (M00529: nitrate => nitrogen). (**D**) Dissimilatory nitrate reduction (M00530: nitrate => ammonia). (**E**) Nitrification (M00528: ammonia => nitrite). (**F**) Assimilatory nitrate reduction (M00531: nitrate => ammonia). The term “1st” indicates the period between the 0th week to the 8th week; “2nd” indicates the period between the 8th week to the 17th week. Red stars indicate the *p*-value of the Mann–Whitney U test < 0.05 (compared with CT). CT: control; BA: benzoic acid; HB: 4-hydroxybenzoate; DH: dehydroacetic acid.

To uncover the effects of BA, HB and DHA on the nitrogen cycle in sediments, six nitrogen cycle associated microbial groups (anaerobic ammonium oxidation (anammox), nitrogen fixation, nitrification, denitrification, dissimilatory nitrate reduction and assimilatory nitrate reduction) were examined. The relative abundance of four nitrogen-cycle-associated microbial groups, anammox (Figure 5A and Figure S4), nitrogen fixation (Figure 5B and Figure S5), denitrification (Figure 5C and Figure S6) and dissimilatory nitrate reduction (Figure 5D and Figure S7) were **increased** after the 8th week in the BA-, HB- and DHA-treated sediments. In contrast, the relative abundance of nitrification-associated microbial communities in the BA-, HB- and DHA-treated sediments were decreased compared with the control sediment (Figure 5E and Figure S8). The assimilatory nitrate reduction microbial communities exhibited no difference between the control and preservative-treated sediments (Figure 5F and Figure S9).

### 2.5. Microbial Community Associated with Sulfur Cycle

**Figure 6:**
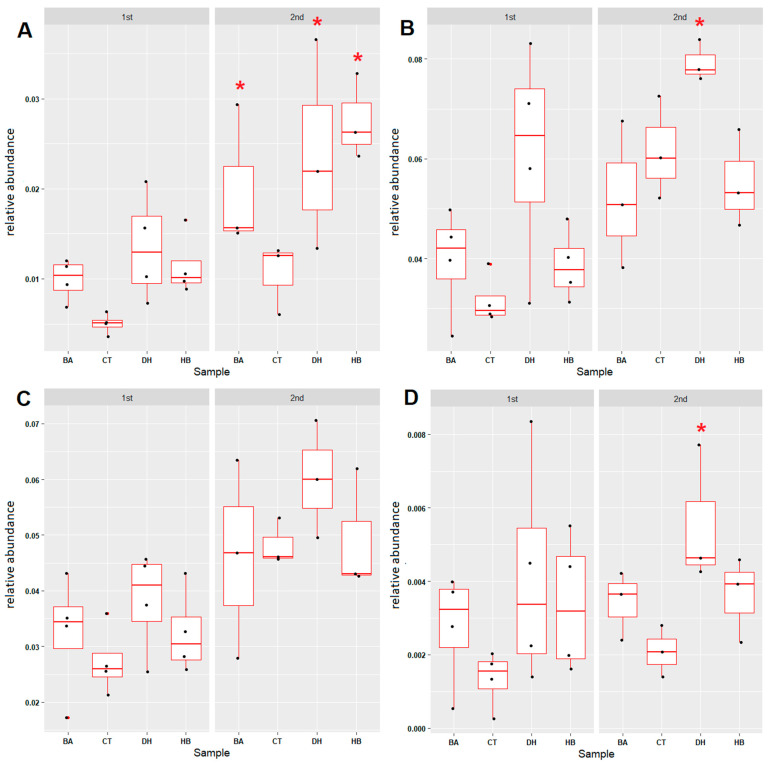
Overall relative abundance changes in sulfur-cycle-associated microbial communities in the BA-, HB- and DHA-treated river sediments. (**A**) Thiosulfate oxidation (M00595: thiosulfate => sulfate). (**B**) Assimilatory sulfate reduction (M00176: sulfate => H_2_S). (**C**) Sulfate-sulfur assimilation (M00616). (**D**) Dissimilatory sulfate reduction (M00596: sulfate => H_2_S). The term “1st” indicates the period between the 0th week to the 8th week; “2nd” indicates the period between the 8th week to the 17th week. Red stars indicate the *p*-value of the Mann–Whitney U test < 0.05 (compared with control (CT)). CT: control; BA: benzoic acid; HB: 4-hydroxybenzoate; DH: dehydroacetic acid.

To reveal the effects of BA, HB and DHA on the sulfur metabolism in sediments, four sulfur-cycle-associated microbial groups (assimilatory sulfate reduction, dissimilatory sulfate reduction, thiosulfate oxidation and sulfate-sulfur assimilation) were examined. The relative abundances of thiosulfate oxidation microbial communities were increased after the eighth week in the BA-, HB- and DHA-treated sediments (Figure 6A and Figure S10). In contrast, the relative abundance of assimilatory sulfate-reduction- (Figure 6B and Figure S11) and dissimilatory sulfate-reduction- (Figure 6D and Figure S13) associated microbial communities in DHA-treated sediments were **increased.** The relative abundances of sulfate-sulfur assimilation microbial communities exhibit no difference between control and preservative-treated sediments (Figure 6C and Figure S12).

### 2.6. Microbial Community Associated with Xenobiotic Degradation and Pathogenic Bacteria

**Figure 7:**
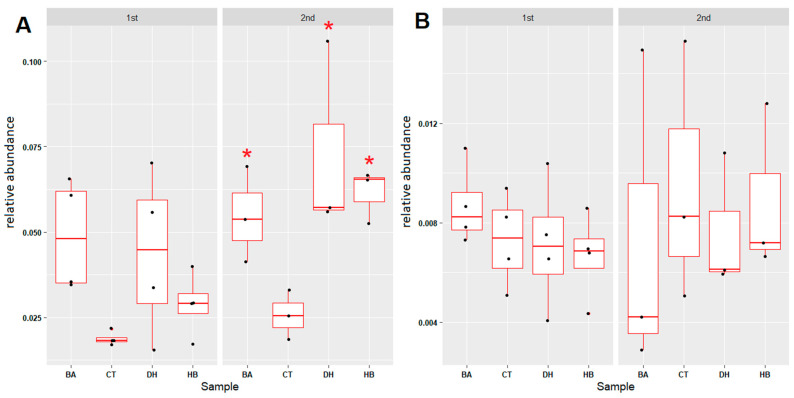
Overall relative abundance changes in microbial communities in the BA-, HB- and DHA-treated river sediments. (**A**) Microbial genera associated with xenobiotic degradation. (**B**) Microbial genera with potential pathogenic bacteria. The term “1st” indicates the period between the 0th week to the 8th week; “2nd” indicates the period between the 8th week to the 17th week. Red stars indicate the *p*-value of the Mann–Whitney U test < 0.05 (compared with control (CT)). CT: control; BA: benzoic acid; HB: 4-hydroxybenzoate; DH: dehydroacetic acid.

**Figure 8:**
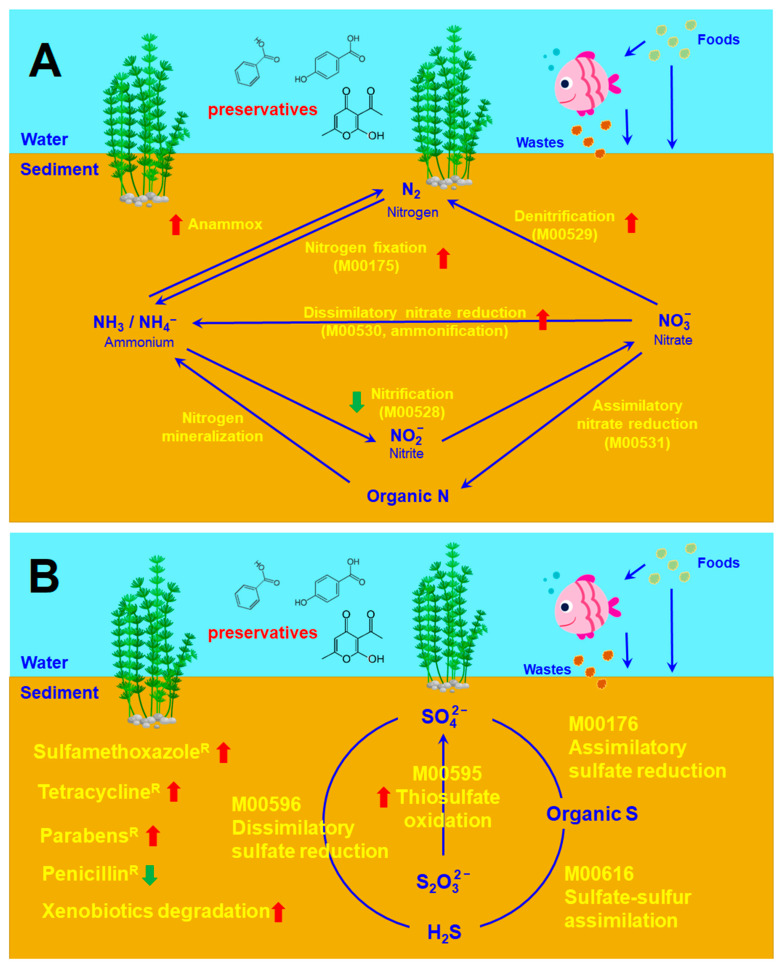
Effects of BA, HB and DHA on microbiomes in freshwater river sediments revealed in this study. (**A**) Nitrogen-cycle-associated microbial communities. (**B**) Sulfur-cycle-associated microbial communities. Red arrows indicate increase in microbes. Green arrows indicate decrease in microbes. “M00xxx” indicates KEGG module ID number.

The relative abundance of xenobiotic-degradation-associated microbial communities were **increased** in all preservative treated sediments (Figure 7A and Figure S14). In contrast, the relative abundance of potential pathogenic bacteria exhibited no difference between control and preservative-treated sediments (Figure 7B and Figure S15). The overall effects of BA, HB and DHA on the microbial communities in freshwater river sediments are summarized in Figure 8.

## 3. Discussions

Results of this study indicate that the three preservatives (BA, HB, and DHA) can promote the increment of sulfonamide-, tetracycline- and preservative-resistant microbes in freshwater river sediments (Figure 1 and Figure 2). Most of the antibiotic- and preservative-resistant microbes in the preservative-treated sediments were increased after the 15th week, suggesting that continuous addition of preservatives may lead to an adaptation/selection pressure in the sediments. The relative abundance of xenobiotic degradation associated microbial communities were increased in all preservative-treated sediments (Figure 7A and Figure S15). Moreover, continuous addition of 20 ppm preservatives every week did not result in an accumulation of high levels of preservatives in the water of fish tanks (Figure S3). These results suggest that the increment of the sulfonamide-, tetracycline- and preservative-resistant microbes may be due to the increment of xenobiotic-degrading microbial communities in sediments.

The profiles of the decrease of penicillin-resistant microbes were not consistent with the profiles of the increment of xenobiotic-degrading microbial communities and the increment of sulfonamide-, tetracycline- and preservative-resistant microbes. One possible explanation for these observations is collateral susceptibility (CS). CS is a phenomenon in which resistance to an antibiotic is associated with susceptibility to another antibiotic. CS occurs not only among different antibiotic groups [27], but also among antibiotics of the same family [28,29]. CS was characterized by identification of antibiotic-resistant strains, then quantifying their sensitivity to other antibiotics [30,31]. CS relationships between antibiotics can be either one-directional or reciprocal. Reciprocal CS exhibits less observed frequency than one-directional CS [32]. In addition to antibiotics, exposure to nonantibiotic conditions, such as heavy metals or biocides, may also lead to reduced sensitivity to antibiotics [33,34]. For example, an adaptation to chlorhexidine (CHX) was shown to be associated with collateral resistance to daptomycin [29]. Antibiotic-resistant strains may exhibit increased sensitivity to antimicrobial peptides [35]. Bacteria growing in media without drugs show decreased antibiotic resistance [36]. Taken together, these studies suggest that CS relationships can occur among antibiotics and nonantibiotic chemicals. Therefore, the co-occurrence of the increase in preservative-resistant microbes and the decrease of penicillin-resistant microbes are reasonable. On the other hand, most of the occurrence of penicillin resistances are due to the horizontal gene transfer of the penicillin-resistant genes (penicillinases) between bacteria [37,38]. Therefore, whether preservatives can reduce/inhibit horizontal gene transfer between bacteria in sediments is worth further study.

There are great effects by the three preservatives on nitrogen- and sulfur-cycle-associated microbial communities in sediments. The ammonium (NH_4_^+^) levels of preservative-treated river water were higher than that of the control river water before the fifth week (Figure 4G). High ammonium (NH_4_^+^) levels in water environments are harmful. Ammonia is toxic to fishes and other aquatic organisms at concentrations below 1 mg/L (ppm) in water [39]. The levels of sulfide (S^2−^), sulfate (SO_4_^2−^), nitrite (NO_2_^−^), nitrate (NO_3_^−^) and ammonium (NH_4_^+^) decreased in the river water of all settings (Figure 3). These results may be due to the incorporation of these inorganic ions into organic compounds (for example, assimilatory nitrate reduction, and assimilatory sulfate reduction). On the other hand, anaerobic ammonium oxidation can proceed with SO_4_^2−^ as a terminal electron acceptor. This process generates N_2_ and S_0_ or HS^−^ as end products. The sulfate-dependent anaerobic ammonium oxidation is also named sulfammox [40]. Sulfammox is a potential microbial process coupling ammonium oxidation with sulfate reduction under anaerobic conditions, which provides a novel link between the nitrogen and sulfur cycle [40,41]. Sulfammox was detected in wastewater treatments and natural environments [42]. Therefore, the composition changes in the sulfate-dependent anaerobic ammonium-oxidizing consortiums by preservatives may lead to ammonium and sulfate removal from water.

The overall effects of BA and HB on microbial communities in sediments are similar, but different from effects of DHA on microbial communities in sediments. This may be due to the similarity of the molecular structures of BA and HB. Moreover, the degradation intermediates of BA and HB in microbes in river sediments are also very similar [43,44].

Although there is a vast amount of studies regarding the distribution of preservatives in aquatic environments, little is known about the effects of preservatives on the biogeochemical processes in aquatic ecosystems. In addition to “ecosystem health”, there is a concern regarding the impact of preservatives on ecosystem services delivered by functional microbes in environments. The chronic exposure effects of preservatives on biogeochemical processes could be performed via the repeated addition of preservatives in the future studies. Moreover, a mixed-batch setup experimental design could be used to mimic aquatic environmental conditions. The tests of the effects of preservatives in sediments are preferably carried out in microcosms or mesocosms. A survey of the preservatives is recommended to rank their ecological impact for regulation of different usages. In addition to the N cycle and S cycle, other biogeochemical processes, such as the carbon cycle or degradation of pollutants, could be considered. The studies investigating the effect of preservatives on biogeochemical processes could be performed by a larger scale, interdisciplinary approach including environmental chemistry and environmental microbiology.

## 4. Materials and Methods

### 4.1. Chemicals

The chemicals benzoic acid (BA), 4-hydroxybenzoate (HB), dehydroacetic acid (DHA), sulfamethoxazole (sul), penicillin (pen), and tetracycline (tet) were purchased from Sigma-Aldrich (Merck/Millipore Sigma, St. Louis, MO, USA). The structure formulae and CAS numbers of these compounds are listed in Tables S3 and S4.

### 4.2. Experimental Design

The river waters and sediments of the Wai-shuangh-si Stream in Taipei city, Taiwan were collected at the sampling site with the GPS coordinates 25.07988, 121.49199. A volume of 10 cm × 45 cm × 45 cm freshwater river sediment and a volume of 30 cm × 45 cm × 45 cm freshwater river water were placed in a 45 cm × 45 cm × 45 cm fish tank with a pump for water circulation (Figure S16A). Four fish tanks for the control, BA, HB and DHA were set up (one preservative per tank). A total of 20 ppm of BA, HB and DHA were added into each tank every week. The timeline of experiment and sampling (for DNA extraction and plate count) is shown in Figure S16B.

### 4.3. Microbial Cultures and Plate Counts

Agar plates composed of 1.5% agar (NEOGEN, Lansing, MI, USA) and 1/3 Tryptic Soy Broth (NEOGEN, USA) were used for total plate count. 1/3 TSA agar plates with 20 μg/mL tetracycline, 50 μg/mL sulfamethoxazole, 100 μg/mL penicillin, or 90 μg/mL of each preservative, were used for plate count of antibiotic- and preservative-resistant microbes. A total of 10 g sediment and 10 mL river water were mixed by vortex. After standing for five minutes, the supernatant was applied to serial dilutions and plate counts. The colonies grown on each plate under 25 °C and aerobic or anaerobic conditions for 3 days were counted.

### 4.4. Analysis of Chemical Compositions in Water

A total of 50 mL for each water sample from fish tanks was firstly filtered using a Whatman 1822-047 GF/C 1.20 µm Glass Microfiber Filters (Whatman, Buckinghamshire, UK) and re-filtered by a 0.22 µm nylon syringe filter (ChromTech, Shanghai, China). The levels of chemical oxygen demand (COD), sulfide (S^2−^), sulfate (SO_4_^2−^), ammonium (NH4^+^), nitrite (NO_2_^−^) and nitrate (NO_3_^−^) were determined by Merck test kits and the Spectroquant Nova 60 photometer (Merck KGaA, Darmstadt, Germany). The pH and ORP of water were analyzed using pH and ORP meters (METTLER TOLEDO, Greifensee, Switzerland).

### 4.5. HPLC Analysis of Residual Preservatives in Water

The water samples were collected and filtered by a 0.22 µm nylon syringe filter (ChromTech, Shanghai, China) and applied to HPLC analysis. The three preservatives were analyzed using an Agilent 1260 HPLC equipped with an InfinityLab PoroShell 120 EC-C18 column and monitored by a photodiode array detector at 254 nm (Agilent Technologies, Inc., Santa Clara, CA, USA). The solvents delivered by the analytical pump were acetonitrile (A) and water (5 mM KH_2_PO_4_) (B). Samples were eluted by 40/60 (A/B) with a flow rate of 1 mL/min. The recovery percentages for BA, HB and DHA were 96.2, 95.3 and 95.6, respectively. The detection limits for the preservatives were 0.1 mg/L.

### 4.6. DNA Extraction, and 16S rRNA Amplicon Sequencing

The PowerSoil DNA Isolation kit (QIAGEN, Venlo, Netherlands) was used for sediment sample DNA extractions. The V5-V8 variable regions of the 16S rRNA gene were amplified. The 5′ primer was composed of 16S rRNA gene-specific sequence 341F (5′-CCTACGGGNBGCASCAG-3′) and sequencing adaptor (5′-TCGTCGGCAGCGTCAGATGTGTATAAGAGACAG3′). The 3′ primer was composed of sequencing adaptor (5′-GTCTCGTGGGCTCGGAGATGTGTATAAGAGACAG3′) and 16S rRNA gene-specific sequence 805R (5′-GACTACNVGGGTATCTAATCC-3′). The PCR reactions were conducted using a 25 µL PCR mixture including PCR buffer, 200 mM of each deoxynucleotide triphosphate, 10 pmol of each primer, 1.25 U of Taq polymerase, and 50 ng of template DNA. The PCR procedures were as follows: 95 °C for 10 min, 30 cycles of 95 °C for 1 min, 55 °C for 1 min, 72 °C for 1 min, and a final step at 72 °C for 15 min. The PCR products were checked by using 1.2% (*w/v*) agarose gel electrophoresis. The 16S rRNA amplicon sequencing was performed using the MiSeq platform (Illumina, Inc., San Diego, CA, USA) at the Cancer Progression Research Center, National Yang Ming Chiao Tung University, Taiwan.

### 4.7. Microbiome Data Analysis

The sequence read trimming was performed using Trimmomatic software (v.0.35). The paired-end reads were merged using FLASH software (v.1.2.11). The chimeric sequences were removed using USEARCH software (v.11). Sequences were grouped into amplicon sequence variants (ASVs) using DADA2 software (v.1.16). ASVs were used to compute the alpha diversity (Shannon, Simpson, Chao 1 and ACE) and beta diversity (NMDS analysis with Bray-Curtis Dissimilarity Distances). The Kruskal–Wallis test was performed to test within-sample group differences for alpha diversity. The metaMDS function in the vegan package of R was used to perform the NMDS analysis. The result of the NMDS analysis was drawn using the ggplot2 package of R. The Analysis of Similarity (ANOSIM) in the vegan package of R was used to evaluate the statistical significance of the beta diversity. Significance was reached while both *p*-value < 0.05 and R value > 0.3 are true. Taxonomic groups (phylum, class, order, family, genus) were assigned using the classifier software from the Ribosomal Database Project (RDP Release 11). Microbial genera associated with the nitrogen cycle, sulfur cycle, xenobiotic degradation and pathogenic bacteria were retrieved from the Kyoto Encyclopedia of Genes and Genomes (KEGG) database [45]. The microbial communities differentially present in the nitrogen cycle, sulfur cycle, xenobiotic degradation and pathogenic bacteria were identified by the Mann–Whitney U test.

For all microbes, *t*-tests of the mean of relative abundances between preservatives and control were performed. The *p* values of the *t*-tests were sorted to produce a list of ranks of mean differences. The top 200 microbes from the list of ranks were retrieved. The plus/minus signs from the differences of means (mean_preservative_ − mean_control_) were used to divide the 200 microbes into “increase” and “decrease” groups. The increased microbes (as well as decreased microbes) from BA, HB and DHA were used to perform the Venn diagram analysis.

## 5. Conclusions

Results of this study reveal that the three preservatives (BA, HB and DHA) have great effects on microbial community compositions in freshwater river sediments. The changes in microbial communities led to the increase in tetracycline-, sulfamethoxazole- and preservative-resistant microbes, and methanogen- and xenobiotic-degradation-associated microbes. Moreover, the three preservatives can also lead to changes in the nitrogen- and sulfur-cycle-associated microbial communities in freshwater river sediments. As a consequence, the three preservatives led to changes in chemical element distributions (efficiencies of assimilation and dissimilation) between organic and inorganic compounds in the river sediments. The effects of the three preservatives on the nitrogen budget, sulfur cycle and methane production in freshwater aquatic environments are worthy of more in-depth investigations.

## Data Availability

The data presented in this study are available in Supplementary Materials.

## Supplementary Materials

The following supporting information can be downloaded at: https://www.mdpi.com/article/10.3390/antibiotics12071082/s1. Figure S1: Total plate counts of microbes in BA-, HB- and DHA-treated sediments, Figure S2: Residual preservatives in each fish tank, Figure S3: Alpha diversities of microbial communities in the BA-, HB- and DHA-treated sediments, Figure S4: Anammox (anaerobic ammonium oxidation) associated microbial communities in the BA-, HB- and DHA-treated sediments, Figure S5: Nitrogen fixation (M00175: nitrogen => ammonia) associated microbial communities in the BA-, HB- and DHA-treated sediments, Figure S6: Denitrification (M00529: nitrate => nitrogen) associated microbial communities in the BA-, HB- and DHA-treated river sediments, Figure S7: Dissimilatory nitrate reduction (M00530: nitrate => ammonia) associated microbial communities in the BA-, HB- and DHA-treated river sediments, Figure S8: Nitrification (M00528: ammonia => nitrite) associated microbial communities in the BA-, HB- and DHA-treated river sediments, Figure S9: Assimilatory nitrate reduction (M00531: nitrate => ammonia) associated microbial communities in the BA-, HB- and DHA-treated river sediments, Figure S10: Thiosulfate oxidation (M00595: thiosulfate => sulfate) associated microbial communities in the BA-, HB- and DHA-treated river sediments, Figure S11: Assimilatory sulfate reduction (M00176: sulfate => H_2_S) associated microbial communities in the BA-, HB- and DHA-treated river sediments, Figure S12: Sulfate-sulfur assimilation (M00616) associated microbial communities in the BA-, HB- and DHA-treated river sediments, Figure S13: Dissimilatory sulfate reduction (M00596: sulfate => H_2_S) associated microbial communities in the BA-, HB- and DHA-treated river sediments, Figure S14: Microbial genera associated with xenobiotics degradation bacteria in the BA-, HB- and DHA-treated river sediments, Figure S15: Proportions of microbial genera with potential pathogenic bacteria in the BA-, HB- and DHA-treated river sediments, Figure S16: Experimental designs, Table S1. The relative abundances (mean ± SD) and *p* values of *t* tests (preservative vs. control) of increased core/shared microbial community, Table S2. The relative abundances (mean ± SD) and *p* values of *t* tests (preservative vs. control) of decreased core/shared microbial community, Table S3: Target compounds used in this study, Table S4. Antibiotics used in this study, EXCLE S1: Microbial communities.
