# Supplementary material for: The Effects of Preservatives on Antibiotic- and Preservative-Resistant Microbes and Nitrogen/Sulfur Cycle Associated Microbial Communities in Freshwater River Sediments
Source: Antibiotics (Basel). 2023 Jun 21;12(7):1082. doi: 10.3390/antibiotics12071082 (PMC10375977; doi:10.3390/antibiotics12071082)
Supplement: Supplementary file 1 [file antibiotics-12-01082-s001.zip › SFigures_STables.pdf]

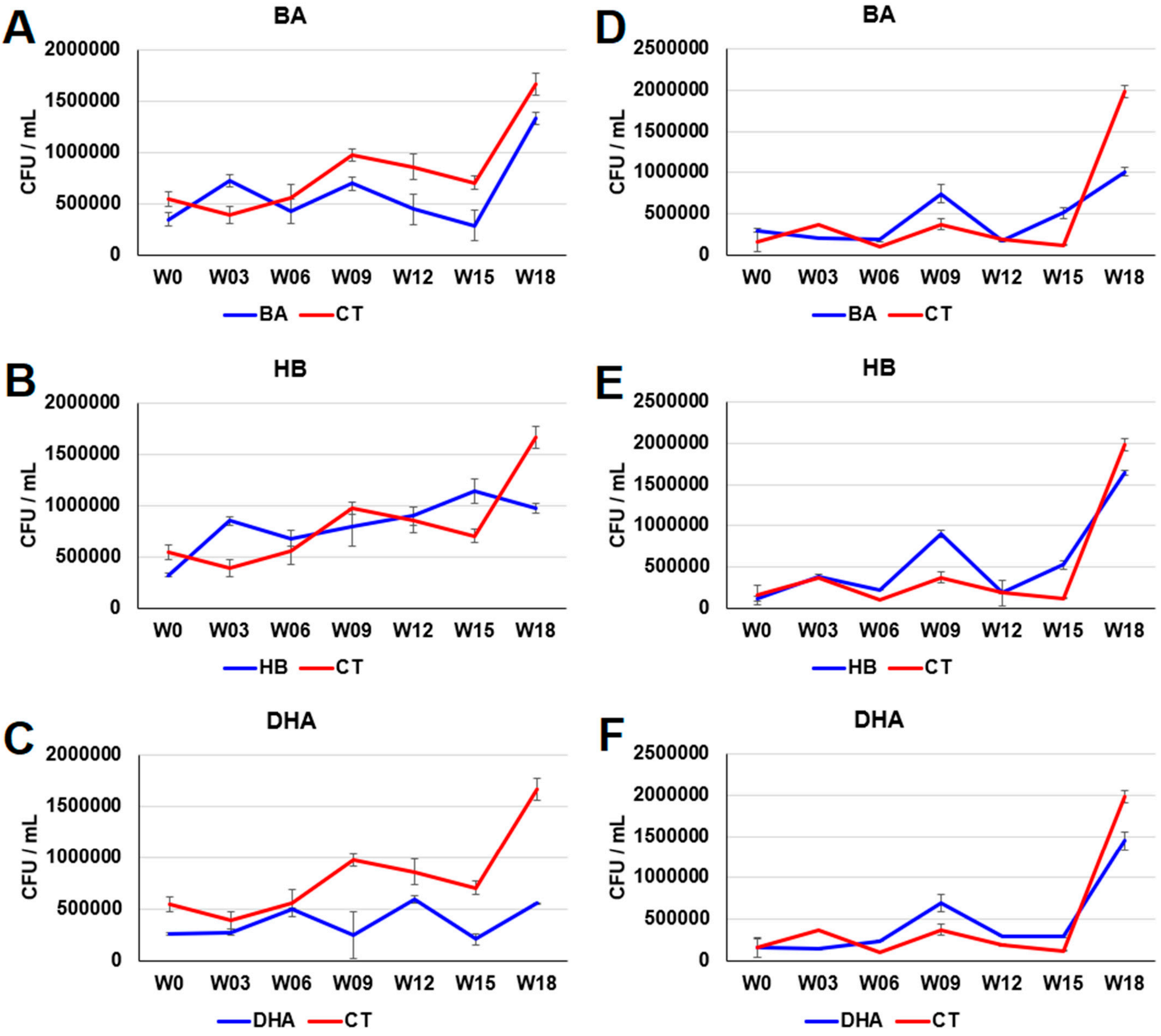

Figure S1. Total plate counts of microbes in BA-, HB- and DHA-treated sediments. A-C: the aerobic cultures. D-F: the anaerobic cultures. Y-axis indicates colony forming unit per mL (CFU/mL). X-axis indicates weeks (0-18th week). Data from triplicate assays are presented as the mean ± SD. CT: control; BA: benzoic acid; HB: 4-hydroxybenzoate; DHA: dehydroacetic acid.

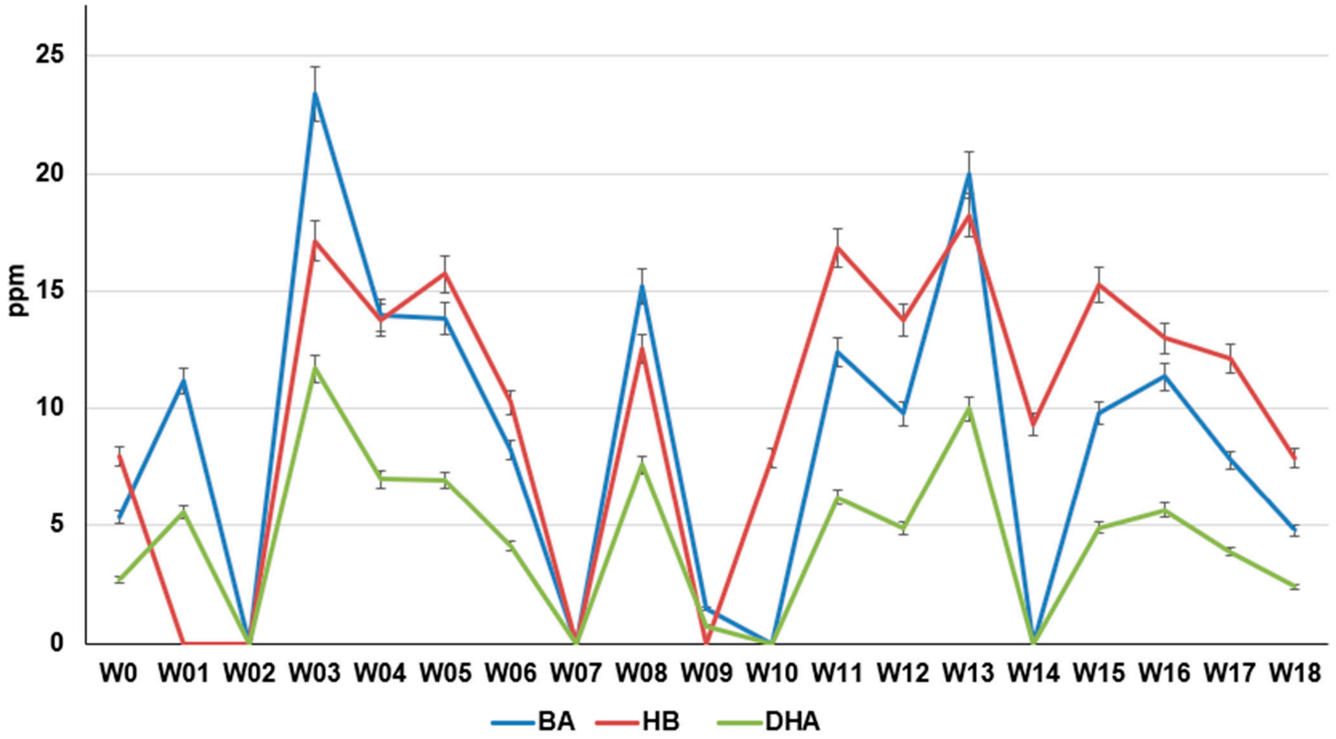

Figure S2. Residual preservatives in each fish tank. Y-axis indicates residual preservatives (parts per million, ppm). X-axis indicates weeks (0-18th week). Data from triplicate assays are presented as the mean ± SD. BA: benzoic acid; HB: 4-hydroxybenzoate; DHA: dehydroacetic acid.

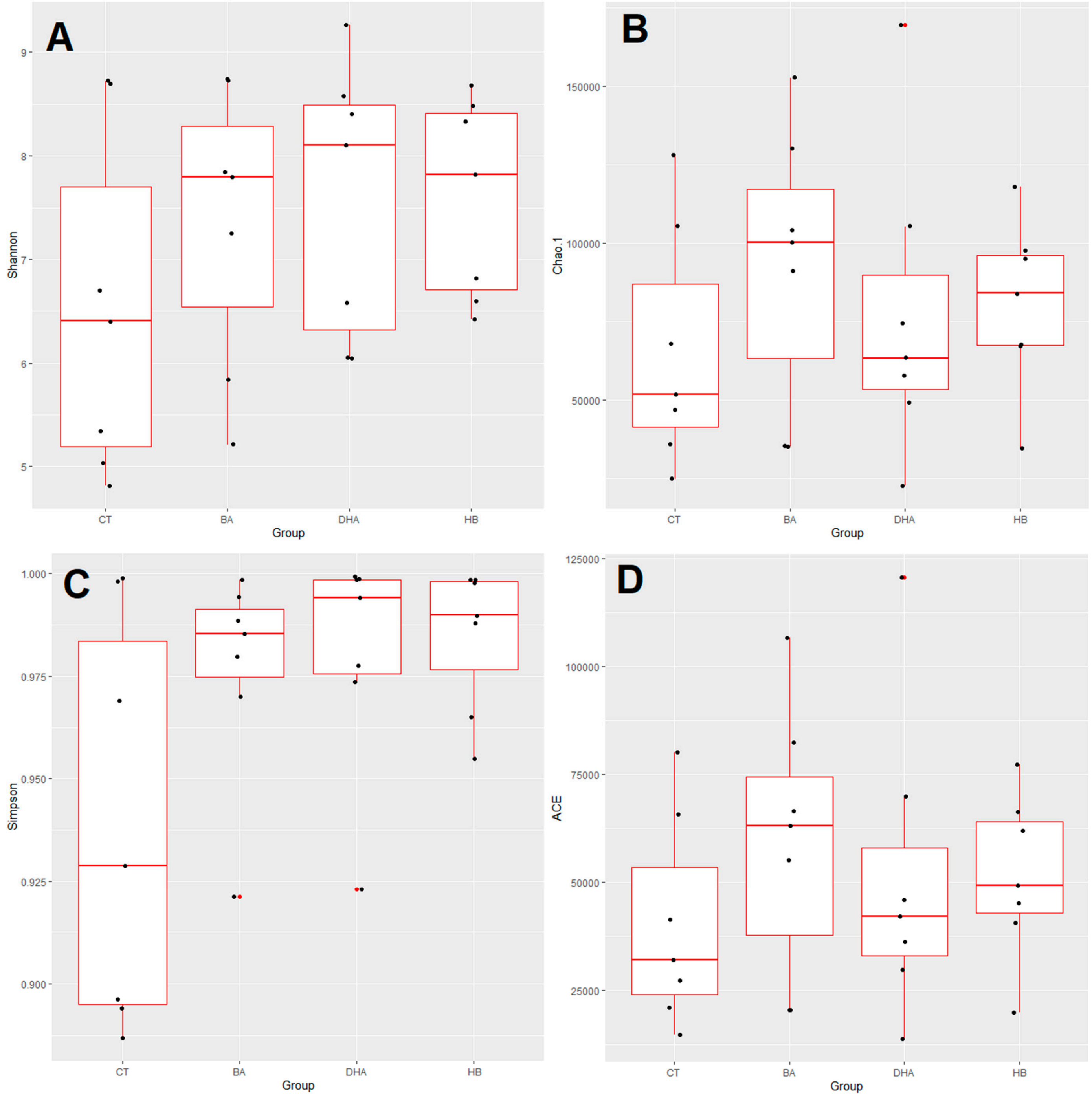

Figure S3. Alpha diversities of microbial communities in the BA-, HB- and DHA-treated sediments. (A) Shannon (B) Chao 1 (C) Simpson (D) ACE. CT: control; BA: benzoic acid; HB: 4-hydroxybenzoate; DHA: dehydroacetic acid.

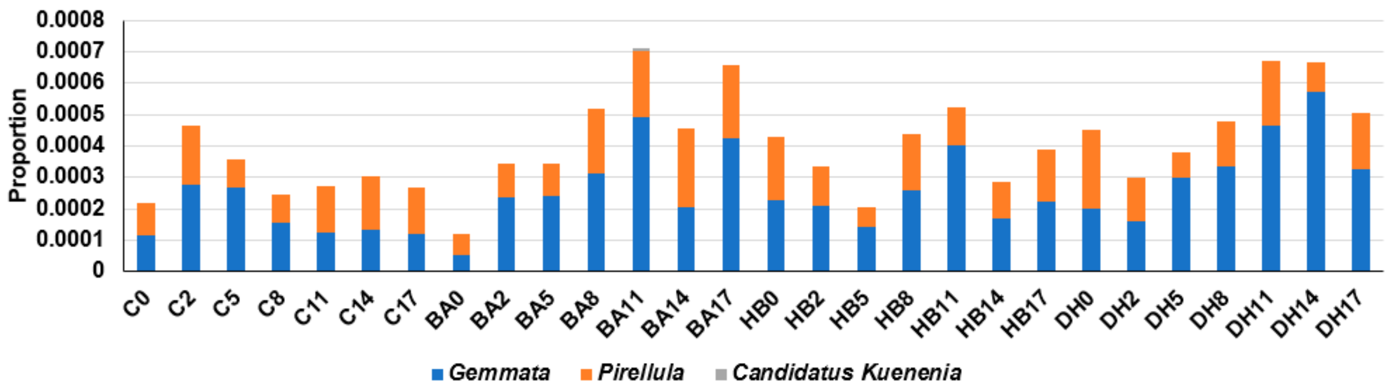

Figure S4. Anammox (anaerobic ammonium oxidation) associated microbial communities in the BA-, HB- and DHA-treated sediments. C: control; BA: benzoic acid; HB: 4-hydroxybenzoate; DH: dehydroacetic acid.

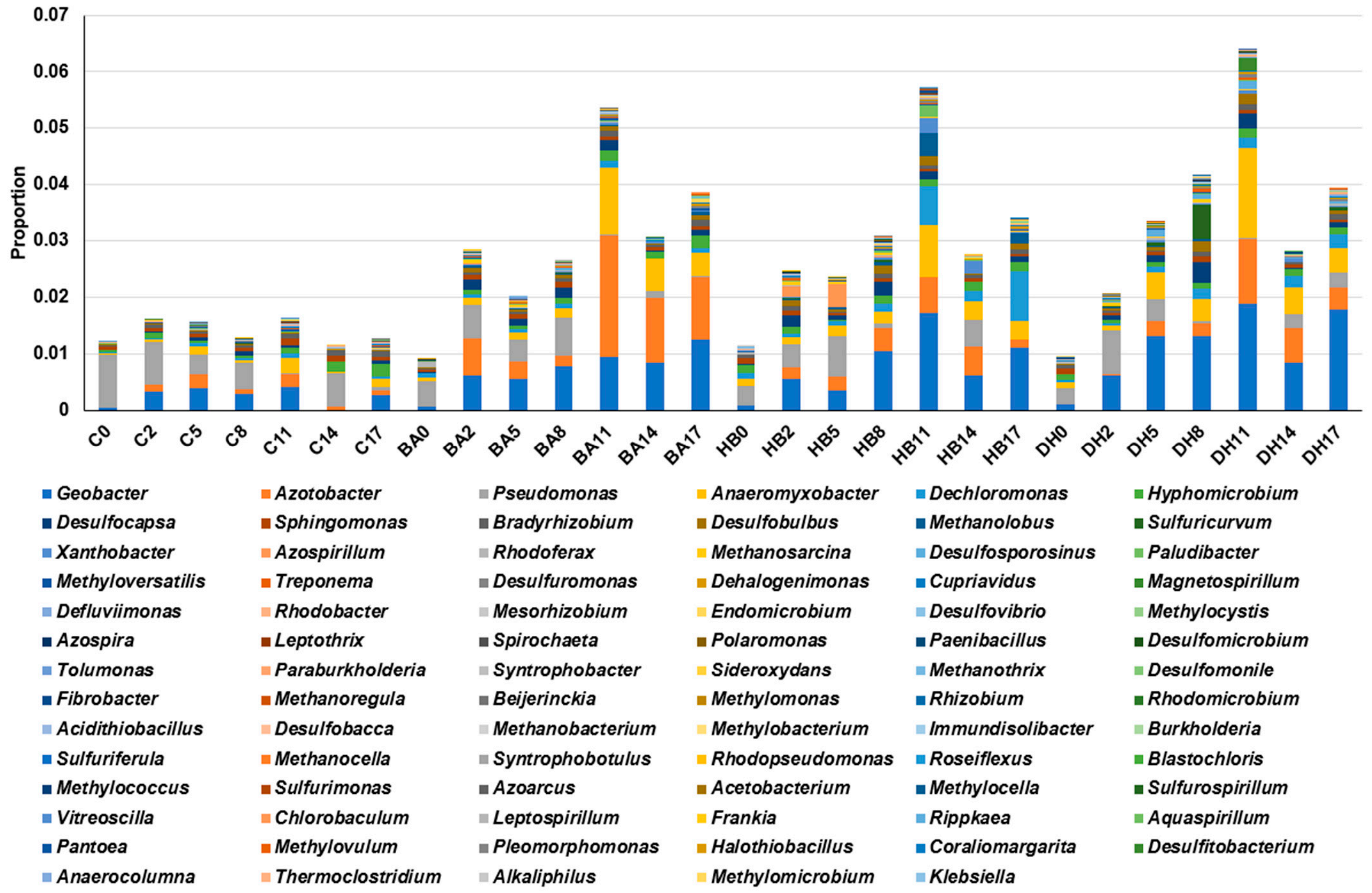

Figure S5. Nitrogen fixation (M00175: nitrogen => ammonia) associated microbial communities in the BA-, HB- and DHA-treated sediments. C: control; BA: benzoic acid; HB: 4-hydroxybenzoate; DH: dehydroacetic acid.

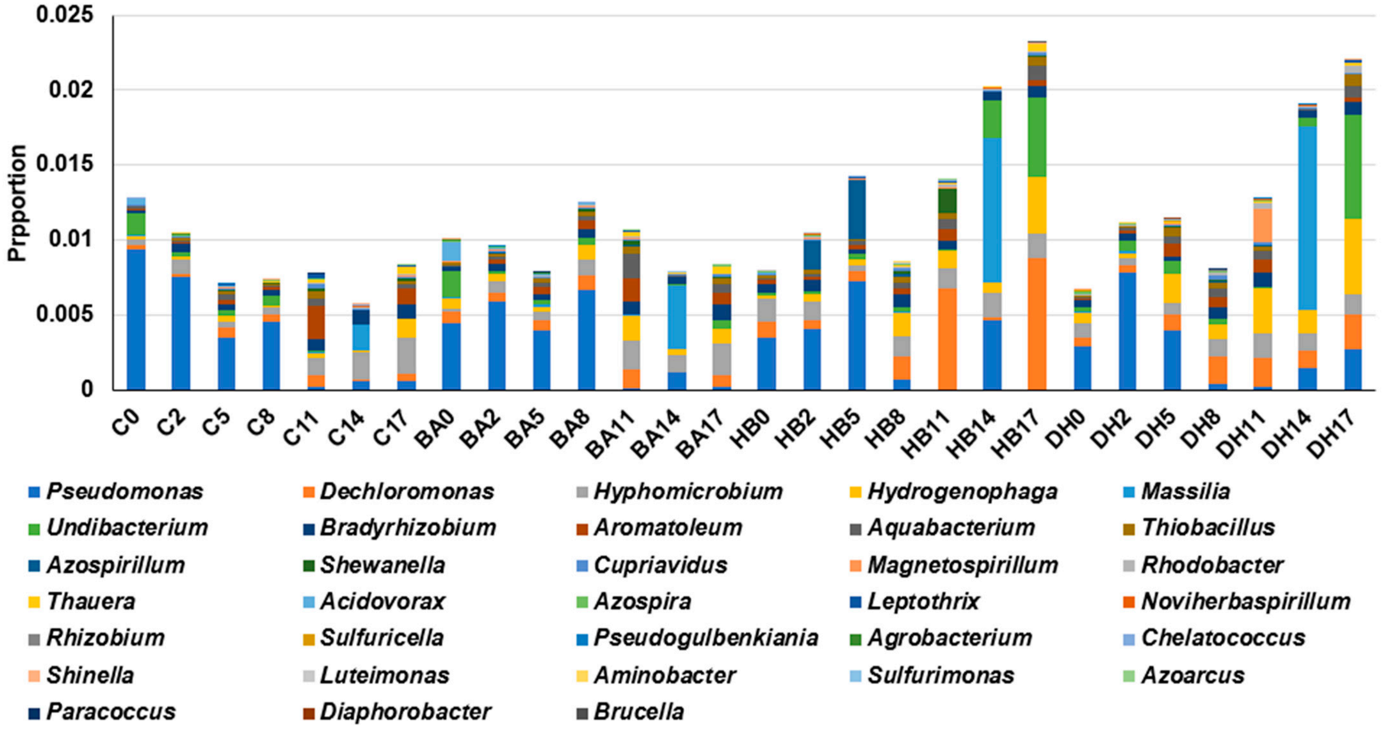

Figure S6. Denitrification (M00529: nitrate => nitrogen) associated microbial communities in the BA-, HB- and DHA-treated sediments. C: control; BA: benzoic acid; HB: 4-hydroxybenzoate; DH: dehydroacetic acid.

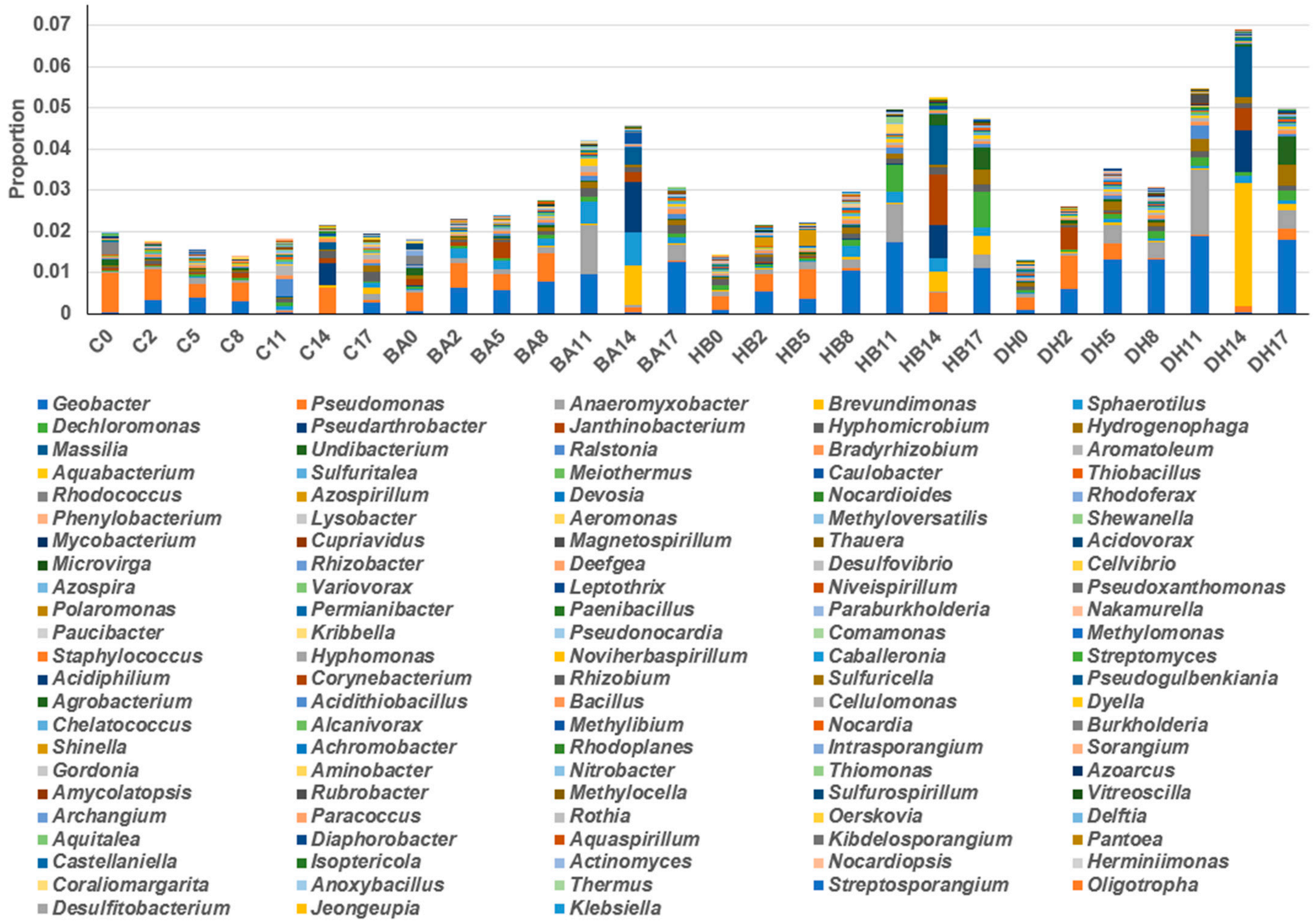

Figure S7. Dissimilatory nitrate reduction (M00530: nitrate => ammonia) associated microbial communities in the BA-, HB- and DHA-treated sediments. C: control; BA: benzoic acid; HB: 4-hydroxybenzoate; DH: dehydroacetic acid.

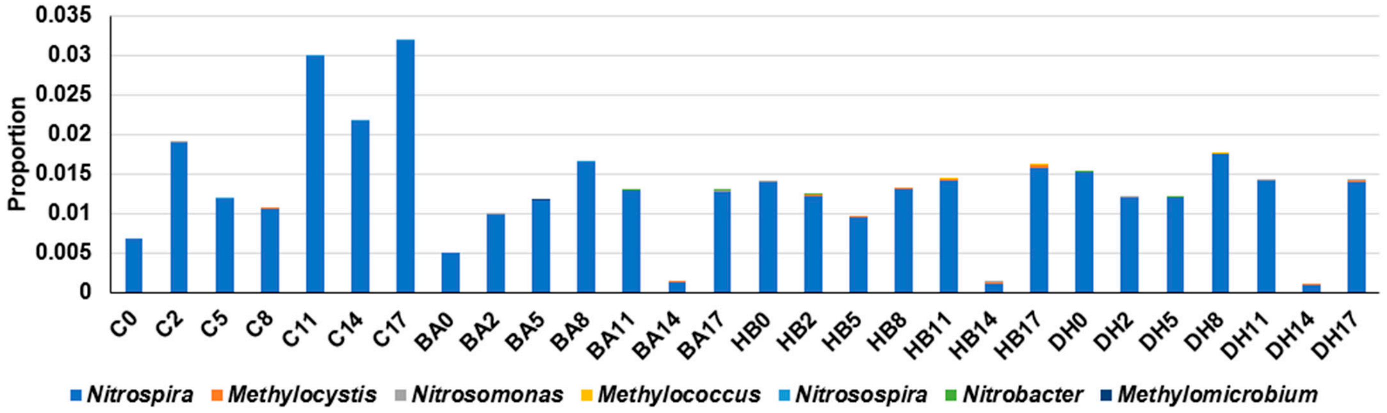

Figure S8. Nitrification (M00528: ammonia => nitrite) associated microbial communities in the BA-, HB- and DHA-treated sediments. C: control; BA: benzoic acid; HB: 4-hydroxybenzoate; DH: dehydroacetic acid.

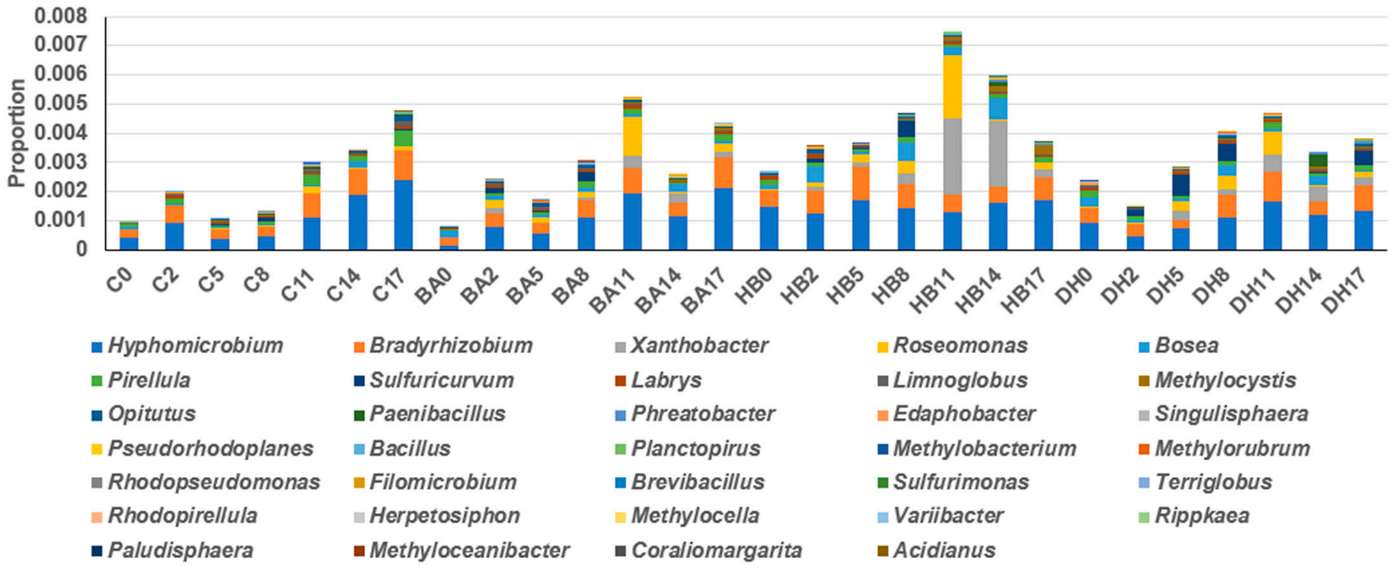

Figure S9. Assimilatory nitrate reduction (M00531: nitrate => ammonia) associated microbial communities in the BA-, HB- and DHA-treated sediments. C: control; BA: benzoic acid; HB: 4-hydroxybenzoate; DH: dehydroacetic acid.

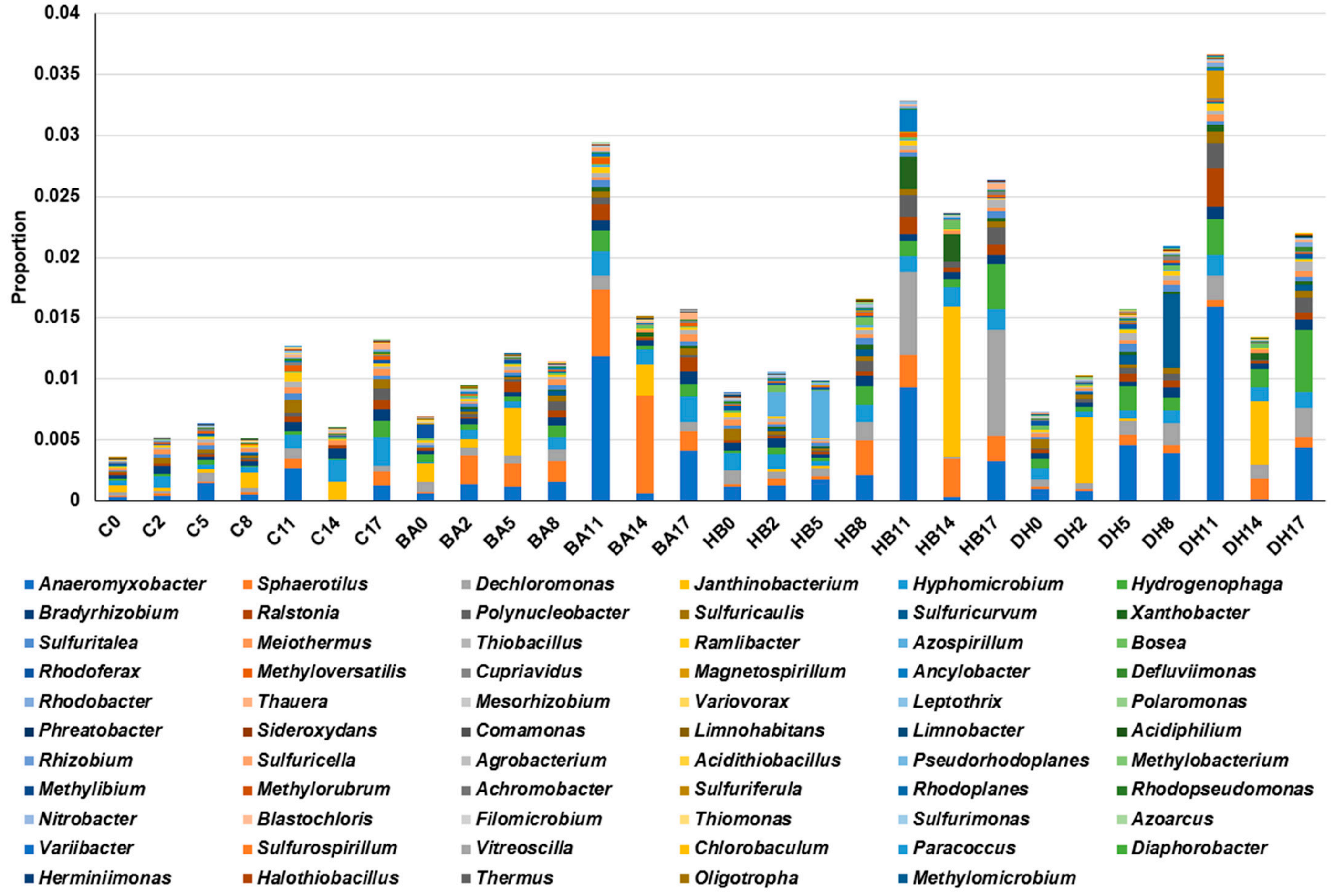

Figure S10. Thiosulfate oxidation (M00595: thiosulfate => sulfate) associated microbial communities in the BA-, HB- and DHA-treated sediments. C: control; BA: benzoic acid; HB: 4-hydroxybenzoate; DH: dehydroacetic acid.

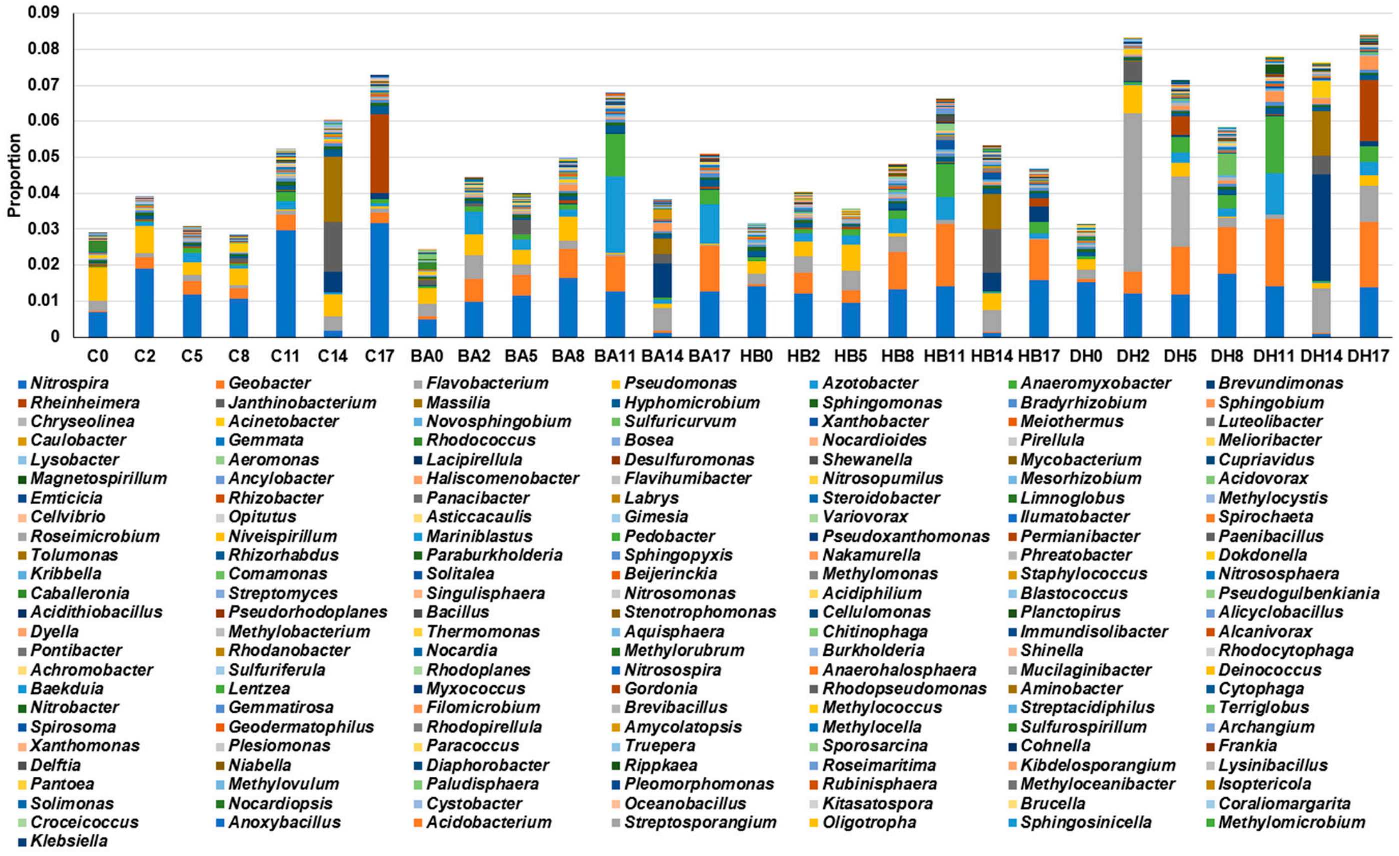

Figure S11. Assimilatory sulfate reduction (M00176: sulfate => H_2S) associated microbial communities in the BA-, HB- and DHA-treated sediments. C: control; BA: benzoic acid; HB: 4-hydroxybenzoate; DH: dehydroacetic acid.

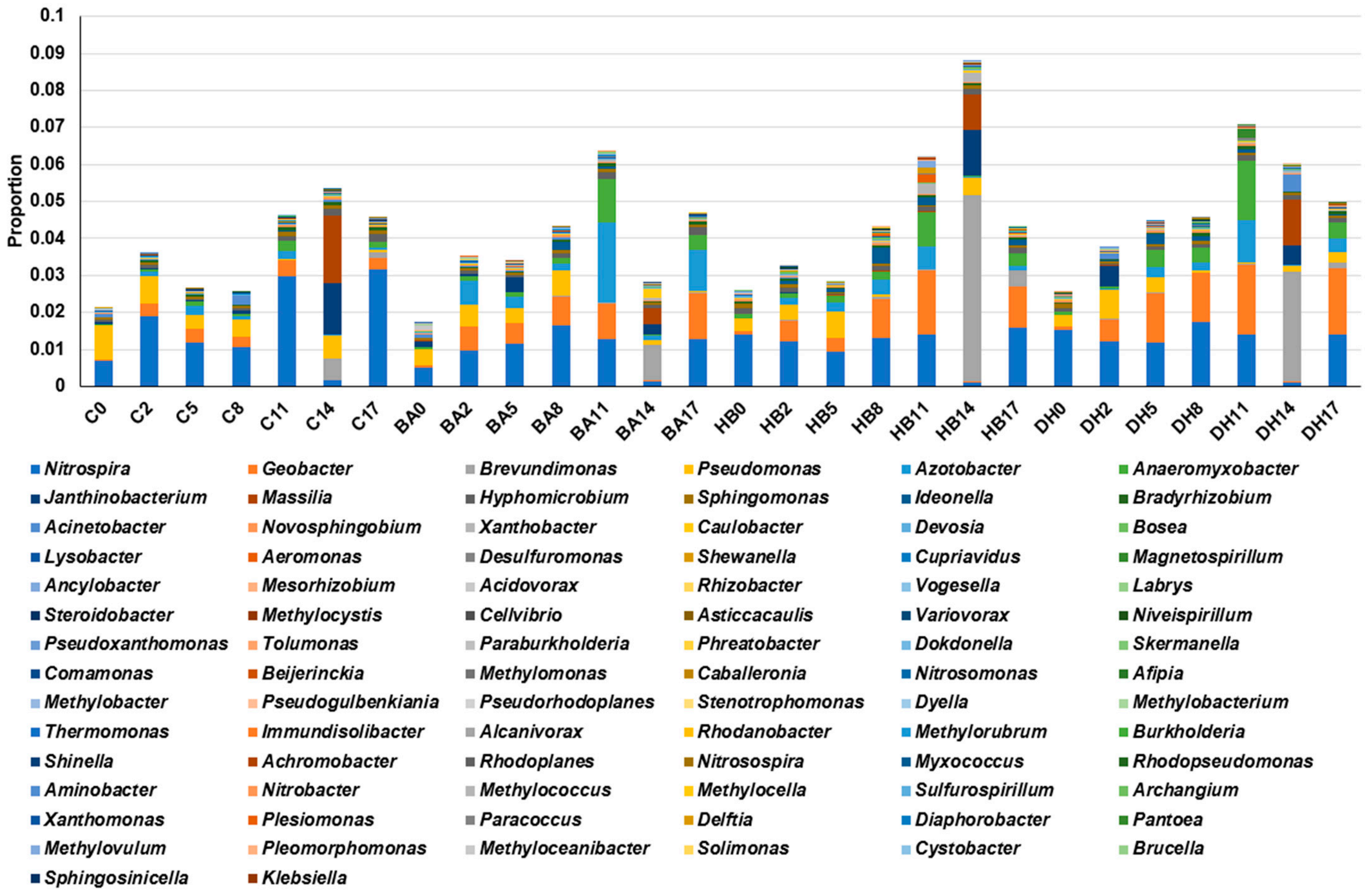

Figure S12. Sulfate-sulfur assimilation (M00616) associated microbial communities in the BA-, HB- and DHA-treated sediments. C: control; BA: benzoic acid; HB: 4-hydroxybenzoate; DH: dehydroacetic acid.

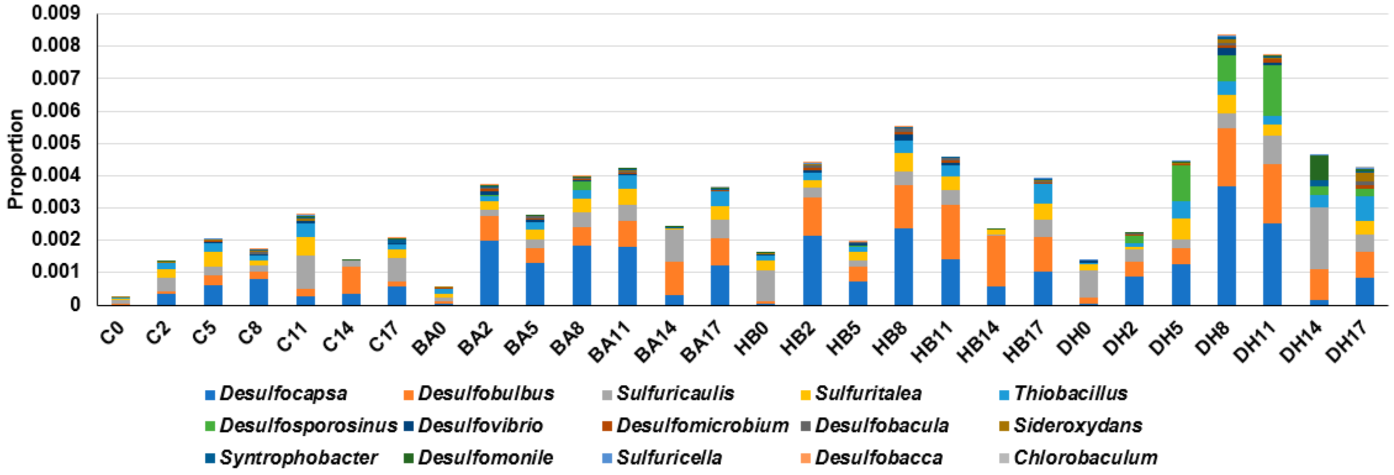

Figure S13. Dissimilatory sulfate reduction (M00596: sulfate => H_2S) associated microbial communities in the BA-, HB- and DHA-treated sediments. C: control; BA: benzoic acid; HB: 4-hydroxybenzoate; DH: dehydroacetic acid.

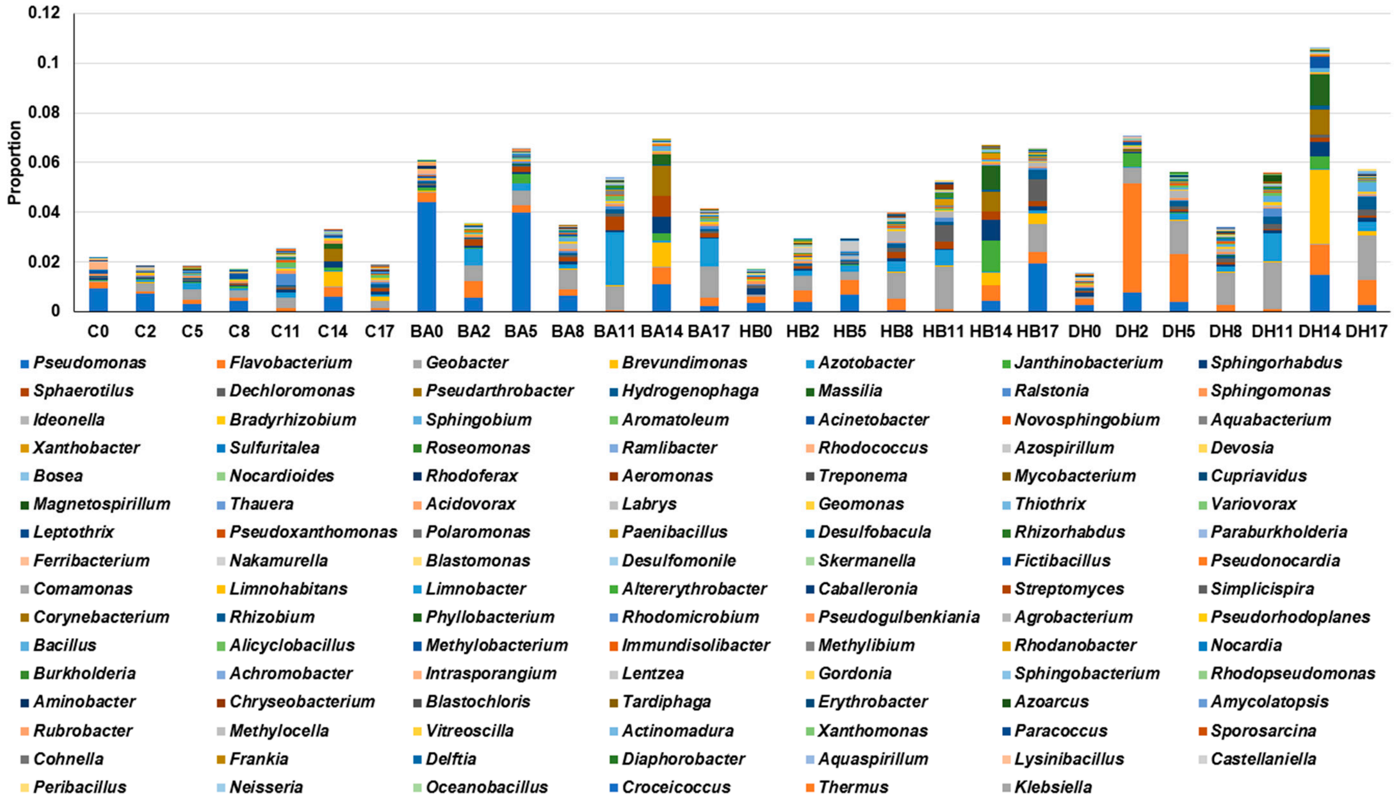

Figure S14. Microbial genera associated with xenobiotics degradation microbes in the BA-, HB- and DHA-treated river sediments. C: control; BA: benzoic acid; HB: 4-hydroxybenzoate; DH: dehydroacetic acid.

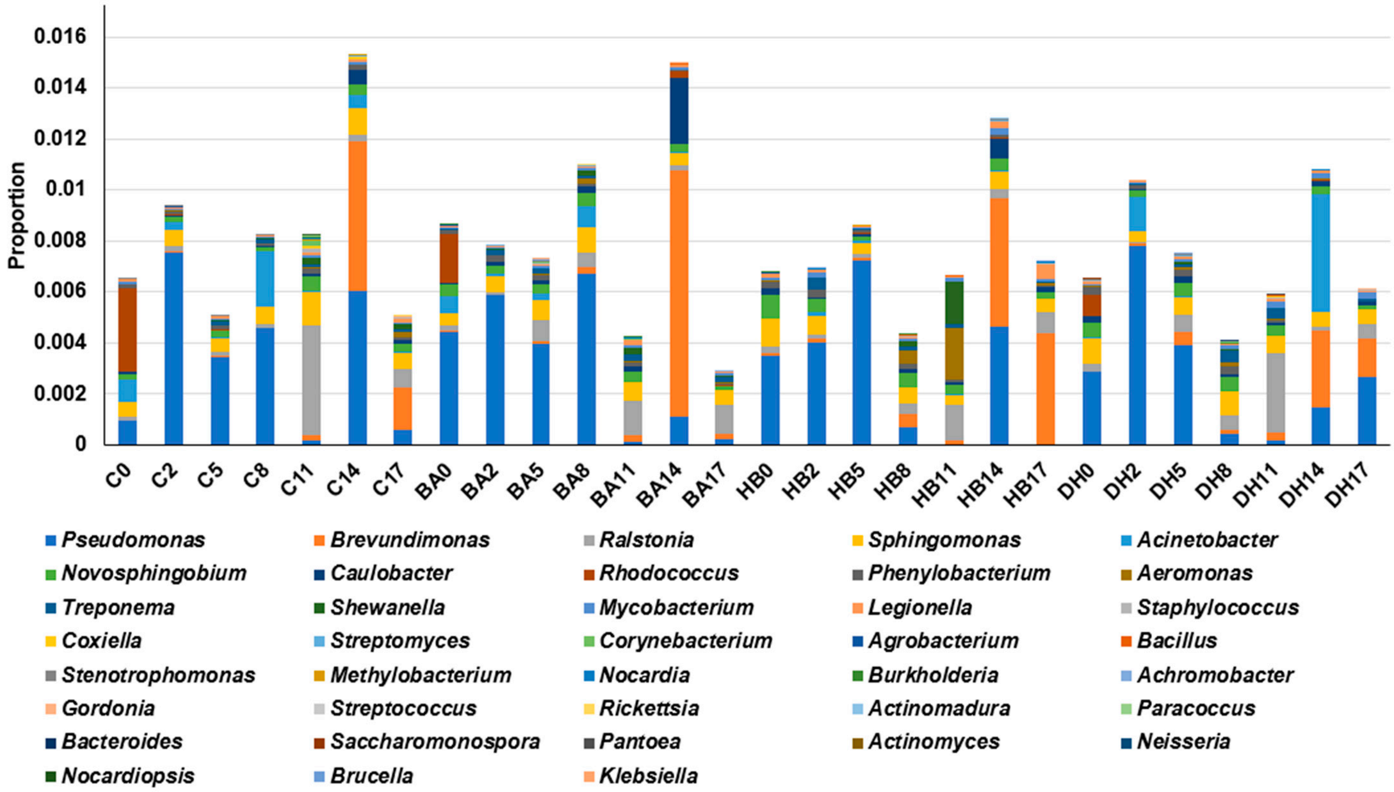

Figure S15. Proportions of microbial genera with potential pathogenic bacteria in the BA-, HB- and DHA-treated river sediments. C: control; BA: benzoic acid; HB: 4-hydroxybenzoate; DH: dehydroacetic acid.

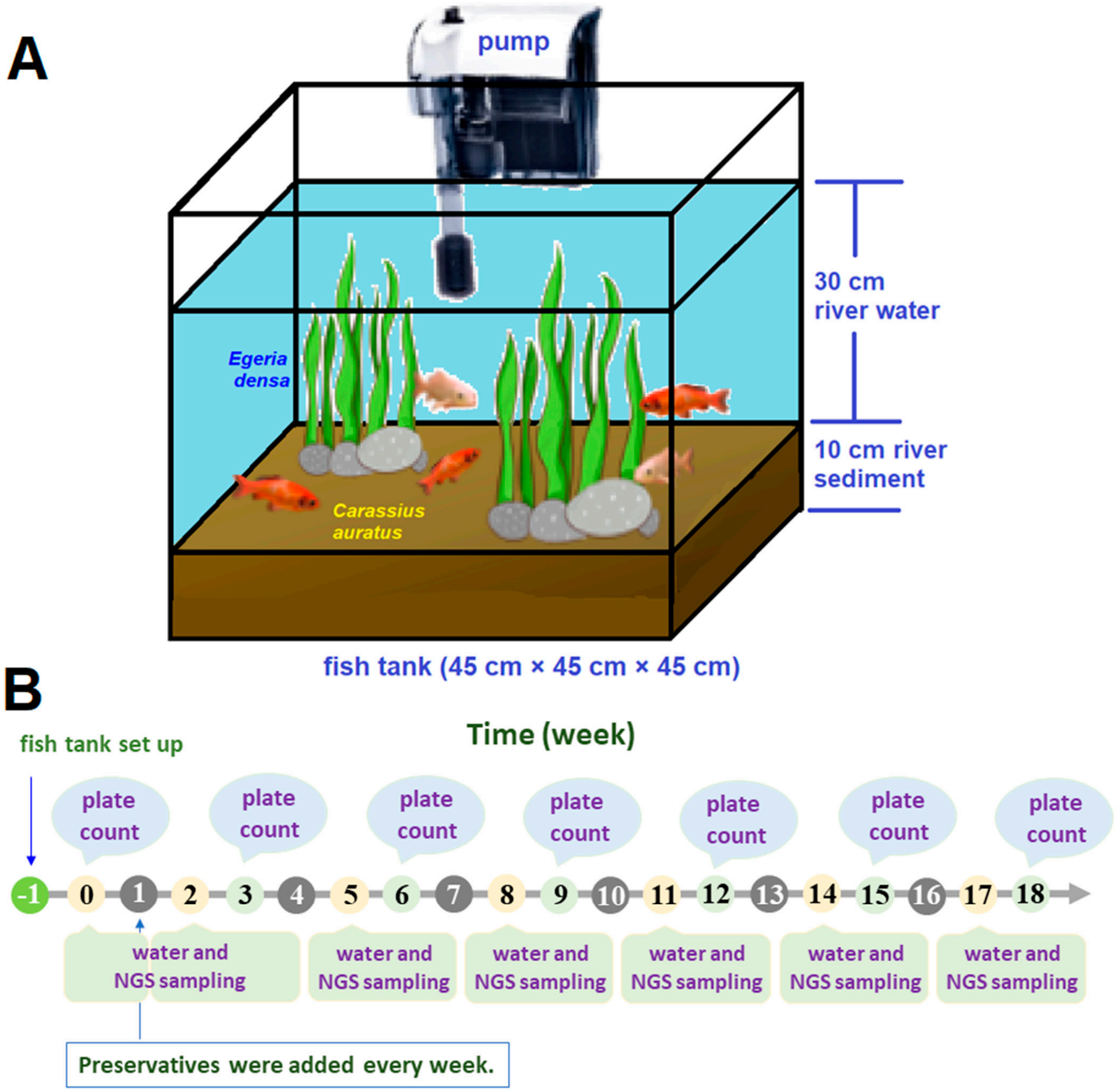

Figure S16. Experimental designs. (A) Settings of fish tanks. (B) Timeline of sampling in this study.

Table S1. The relative abundances (mean ± SD) and p values of t tests (preservative vs control) of increased core/shared microbial community.

Genus	Control	BA	p value	HB	p value	HDA	p value
Alicyclobacillus	0.00E+00 ± 0.00E+00	1.35E-05 ± 1.55E-05	3.95E-02	7.31E-06 ± 7.71E-06	2.75E-02	5.59E-06 ± 9.55E-06	1.47E-01
Amaricoccus	3.03E-05 ± 3.01E-05	1.31E-04 ± 1.81E-04	1.74E-01	2.06E-04 ± 2.42E-04	8.07E-02	8.47E-05 ± 9.39E-05	1.70E-01

Anaeromyxobacter	9.49E-04 ± 9.35E-04	3.03E-03 ± 4.09E-03	2.13E-01	2.76E-03 ± 3.01E-03	1.56E-01	4.39E-03 ± 5.42E-03	1.23E-01
Ancylobacter	8.31E-06 ± 1.06E-05	7.21E-05 ± 7.94E-05	5.68E-02	2.99E-04 ± 6.73E-04	2.76E-01	4.08E-05 ± 6.53E-05	2.18E-01
Azospirillum	7.11E-06 ± 7.04E-06	3.86E-05 ± 5.61E-05	1.66E-01	8.62E-04 ± 1.52E-03	1.62E-01	2.05E-05 ± 3.18E-05	2.96E-01
Derxia	1.66E-06 ± 4.40E-06	8.38E-05 ± 8.72E-05	2.85E-02	1.10E-03 ± 1.13E-03	2.40E-02	1.13E-04 ± 2.48E-04	2.57E-01
Desulfobulbus	1.56E-04 ± 1.03E-04	5.21E-04 ± 3.18E-04	1.34E-02	8.50E-04 ± 6.27E-04	1.36E-02	8.08E-04 ± 7.14E-04	3.40E-02
Desulfomicrobium	1.59E-06 ± 4.20E-06	3.05E-05 ± 2.37E-05	7.82E-03	3.98E-05 ± 3.64E-05	1.73E-02	3.78E-05 ± 3.78E-05	2.68E-02
Gemmobacter	2.15E-04 ± 2.00E-04	3.37E-04 ± 2.53E-04	3.38E-01	3.88E-04 ± 2.63E-04	1.93E-01	4.88E-04 ± 3.53E-04	1.00E-01
Hydrogenophaga	3.61E-04 ± 4.18E-04	7.87E-04 ± 4.72E-04	9.91E-02	1.17E-03 ± 1.21E-03	1.21E-01	1.94E-03 ± 1.63E-03	2.84E-02
Ideonella	9.37E-05 ± 1.07E-04	4.31E-04 ± 7.90E-04	2.85E-01	1.40E-03 ± 1.42E-03	3.18E-02	7.99E-04 ± 1.04E-03	1.00E-01
Luteibacter	0.00E+00 ± 0.00E+00	4.03E-06 ± 5.08E-06	5.79E-02	4.25E-06 ± 7.85E-06	1.77E-01	6.59E-06 ± 9.49E-06	9.14E-02
Maribellus	3.22E-05 ± 2.85E-05	1.87E-04 ± 1.39E-04	1.36E-02	3.30E-04 ± 4.95E-04	1.38E-01	2.08E-04 ± 2.01E-04	4.06E-02
Methanolobus	0.00E+00 ± 0.00E+00	2.36E-04 ± 2.38E-04	2.21E-02	1.02E-03 ± 1.50E-03	9.86E-02	1.03E-04 ± 1.92E-04	1.79E-01
Methanomethylovorans	0.00E+00 ± 0.00E+00	1.76E-03 ± 1.02E-03	6.45E-04	4.08E-03 ± 4.71E-03	4.06E-02	6.72E-04 ± 8.62E-04	6.15E-02
Methanoregula	0.00E+00 ± 0.00E+00	2.66E-05 ± 6.65E-05	3.11E-01	2.28E-05 ± 3.27E-05	9.05E-02	9.89E-06 ± 1.38E-05	8.19E-02

Methanosarcina	1.37E-05 ± 1.45E-05	1.97E-04 ± 2.31E-04	5.76E-02	2.75E-04 ± 2.63E-04	2.20E-02	2.19E-04 ± 2.60E-04	5.98E-02
Methylobacter	1.66E-06 ± 4.40E-06	1.04E-05 ± 9.90E-06	5.32E-02	1.34E-05 ± 1.53E-05	7.52E-02	8.61E-06 ± 1.79E-05	3.39E-01
Oharaeibacter	1.28E-06 ± 3.39E-06	1.14E-05 ± 2.34E-05	2.78E-01	3.72E-05 ± 4.02E-05	3.62E-02	1.61E-05 ± 2.38E-05	1.29E-01
Phreatobacter	2.69E-06 ± 4.60E-06	1.88E-05 ± 2.73E-05	1.49E-01	2.92E-05 ± 3.23E-05	5.25E-02	3.42E-05 ± 3.67E-05	4.41E-02
Pinisolibacter	0.00E+00 ± 0.00E+00	2.96E-05 ± 4.89E-05	1.36E-01	6.99E-05 ± 7.69E-05	3.31E-02	4.96E-05 ± 8.57E-05	1.52E-01
Prosthecobacter	6.17E-05 ± 6.50E-05	2.74E-04 ± 1.98E-04	1.95E-02	2.88E-04 ± 2.69E-04	5.13E-02	2.06E-04 ± 2.24E-04	1.28E-01
Prosthecomicrobium	4.21E-05 ± 4.10E-05	1.26E-04 ± 8.95E-05	4.34E-02	1.61E-04 ± 1.07E-04	1.82E-02	2.38E-04 ± 2.00E-04	2.62E-02
Roseomonas	7.18E-05 ± 7.54E-05	3.28E-04 ± 4.59E-04	1.72E-01	4.83E-04 ± 7.62E-04	1.81E-01	2.59E-04 ± 2.89E-04	1.24E-01
Sediminicoccus	1.28E-06 ± 3.39E-06	1.38E-05 ± 1.86E-05	1.05E-01	1.56E-05 ± 1.23E-05	1.15E-02	2.47E-05 ± 2.88E-05	5.40E-02
Subdivision5	2.37E-05 ± 2.44E-05	3.44E-04 ± 2.72E-04	9.21E-03	5.29E-04 ± 2.77E-04	4.26E-04	3.46E-04 ± 3.34E-04	2.56E-02
Terrimicrobium	8.46E-05 ± 1.69E-04	8.88E-04 ± 1.06E-03	7.16E-02	6.31E-04 ± 8.02E-04	1.03E-01	4.33E-04 ± 5.64E-04	1.43E-01
Thermostilla	4.53E-06 ± 5.71E-06	2.02E-05 ± 2.70E-05	1.59E-01	3.52E-05 ± 3.49E-05	4.09E-02	1.25E-05 ± 2.30E-05	3.92E-01
Tolumonas	2.87E-06 ± 4.94E-06	6.08E-05 ± 1.06E-04	1.76E-01	1.99E-05 ± 2.69E-05	1.24E-01	2.12E-05 ± 2.68E-05	1.01E-01
Victivallis	0.00E+00 ± 0.00E+00	9.32E-06 ± 9.53E-06	2.37E-02	2.31E-04 ± 2.69E-04	4.18E-02	6.50E-05 ± 6.61E-05	2.31E-02

Xanthobacter	0.00E+00 ± 0.00E+00	1.78E-04 ± 1.56E-04	1.05E-02	8.28E-04 ± 1.10E-03	7.03E-02	2.65E-04 ± 2.29E-04	9.83E-03

Table S2. The relative abundances (mean ± SD) and p values of t tests (preservative vs control) of decreased core/shared microbial community.

Genus	Control	BA	p value	HB	p value	HDA	p value
Acanthopleuribacter	4.61E-06 ± 8.88E-06	0.00E+00 ± 0.00E+00	1.95E-01	0.00E+00 ± 0.00E+00	1.95E-01	0.00E+00 ± 0.00E+00	1.95E-01
Aridibacter	3.31E-04 ± 2.36E-04	1.68E-04 ± 1.03E-04	1.18E-01	1.41E-04 ± 7.88E-05	6.58E-02	1.92E-04 ± 9.88E-05	1.74E-01
Bacillariophyta	9.12E-04 ± 6.54E-04	6.03E-04 ± 2.19E-04	2.58E-01	5.71E-04 ± 3.39E-04	2.45E-01	7.58E-04 ± 3.99E-04	6.06E-01
Cellvibrio	1.05E-04 ± 2.00E-04	1.52E-05 ± 1.46E-05	2.60E-01	2.13E-05 ± 3.26E-05	2.97E-01	3.98E-05 ± 4.61E-05	4.19E-01
Chujaibacter	7.57E-06 ± 7.75E-06	0.00E+00 ± 0.00E+00	2.39E-02	0.00E+00 ± 0.00E+00	2.39E-02	0.00E+00 ± 0.00E+00	2.39E-02
Comamonas	3.49E-05 ± 2.28E-05	1.61E-05 ± 1.64E-05	1.03E-01	9.50E-06 ± 1.81E-05	3.95E-02	1.77E-06 ± 4.69E-06	2.69E-03
Deinococcus	1.02E-05 ± 1.39E-05	0.00E+00 ± 0.00E+00	7.58E-02	0.00E+00 ± 0.00E+00	7.58E-02	2.73E-06 ± 7.21E-06	2.30E-01
Flavihumibacter	1.53E-04 ± 1.61E-04	5.95E-05 ± 4.28E-05	1.64E-01	4.48E-05 ± 3.66E-05	1.09E-01	1.20E-04 ± 8.94E-05	6.50E-01
Gp9	8.17E-05 ± 7.09E-05	4.88E-05 ± 1.06E-05	2.48E-01	4.02E-05 ± 3.82E-05	1.97E-01	4.48E-05 ± 3.93E-05	2.51E-01
Gp15	6.02E-05 ± 5.83E-05	2.51E-05 ± 1.54E-05	1.50E-01	3.39E-05 ± 1.58E-05	2.72E-01	1.49E-05 ± 1.59E-05	7.07E-02
Gp20	1.70E-05 ± 1.91E-05	5.44E-06 ± 7.48E-06	1.64E-01	2.68E-06 ± 4.58E-06	7.90E-02	2.73E-06 ± 7.21E-06	9.05E-02
Inhella	8.29E-05 ± 6.46E-	5.03E-05 ± 5.38E-	3.26E-01	7.77E-05 ± 8.29E-	8.98E-01	5.10E-05 ± 5.93E-	3.54E-01

	05	05		05		05	
Kribbella	2.16E-05 ± 6.27E-06	1.72E-05 ± 9.57E-06	3.28E-01	1.20E-05 ± 1.27E-05	9.87E-02	1.21E-05 ± 1.40E-05	1.29E-01
Litorilinea	1.71E-04 ± 1.01E-04	9.45E-05 ± 5.54E-05	1.05E-01	1.24E-04 ± 4.82E-05	2.91E-01	1.38E-04 ± 1.00E-04	5.59E-01
Methylotenera	3.03E-02 ± 3.65E-02	1.32E-02 ± 1.66E-02	2.83E-01	1.97E-02 ± 1.59E-02	4.96E-01	8.45E-03 ± 1.24E-02	1.60E-01
Methylovorus	1.63E-04 ± 4.27E-04	5.35E-05 ± 1.12E-04	5.23E-01	7.21E-05 ± 1.68E-04	6.09E-01	1.66E-04 ± 3.06E-04	9.90E-01
Nitrosospira	6.96E-06 ± 6.94E-06	1.35E-06 ± 3.56E-06	8.14E-02	0.00E+00 ± 0.00E+00	2.11E-02	5.26E-06 ± 9.04E-06	7.01E-01
Oryzihumus	6.96E-06 ± 6.94E-06	2.42E-06 ± 4.14E-06	1.63E-01	3.20E-06 ± 5.75E-06	2.91E-01	1.67E-06 ± 4.42E-06	1.15E-01
Parafilimonas	5.00E-05 ± 2.92E-05	2.52E-05 ± 1.66E-05	7.43E-02	2.19E-05 ± 1.87E-05	5.36E-02	2.07E-05 ± 2.08E-05	5.17E-02
Rhizobium	1.59E-05 ± 1.75E-05	8.00E-06 ± 6.14E-06	2.85E-01	4.82E-06 ± 9.68E-06	1.71E-01	7.54E-06 ± 1.35E-05	3.40E-01
Rhodomicrobium	1.60E-05 ± 1.13E-05	5.33E-06 ± 1.02E-05	8.87E-02	5.17E-06 ± 8.90E-06	6.99E-02	8.14E-06 ± 1.42E-05	2.75E-01
Schlesneria	1.29E-04 ± 1.25E-04	5.64E-05 ± 2.59E-05	1.58E-01	7.38E-05 ± 4.32E-05	2.91E-01	5.66E-05 ± 4.80E-05	1.77E-01
Silvanigrella	7.59E-06 ± 8.54E-06	0.00E+00 ± 0.00E+00	3.66E-02	0.00E+00 ± 0.00E+00	3.66E-02	2.40E-06 ± 6.35E-06	2.21E-01
Thermomonas	1.25E-05 ± 8.12E-06	2.88E-06 ± 4.93E-06	2.00E-02	7.00E-06 ± 1.07E-05	3.00E-01	1.37E-06 ± 3.62E-06	6.15E-03
Truepera	2.87E-06 ± 4.94E-06	0.00E+00 ± 0.00E+00	1.50E-01	1.15E-06 ± 3.05E-06	4.49E-01	0.00E+00 ± 0.00E+00	1.50E-01
Turneriella	5.73E-05 ± 5.64E-	2.07E-05 ± 1.81E-	1.28E-01	2.19E-05 ± 2.60E-	1.57E-01	2.03E-05 ± 2.79E-	1.45E-01

	05	05		05		05	
Variovorax	5.43E-05 ± 1.83E-05	4.31E-05 ± 4.48E-05	5.52E-01	2.20E-05 ± 2.06E-05	9.00E-03	2.87E-05 ± 2.47E-05	4.69E-02

Table S3. Target compounds used in this study.

Target Compounds	abbreviation	CAS No	Formula
Benzoic acid	BA	65-85-0	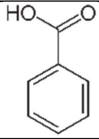
4-hydroxybenzoate	HB	99-96-7	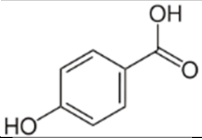
Dehydroacetic acid	DHA	520-45-6	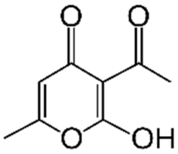

Table S4. Antibiotics used in this study.

Antibiotics	abbreviation	CAS No	Formula
Penicillin	Pen	113-98-4	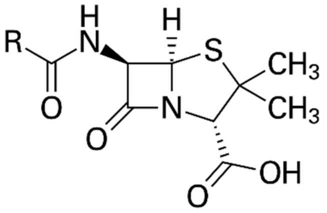
Tetracycline	Tet	60-54-8	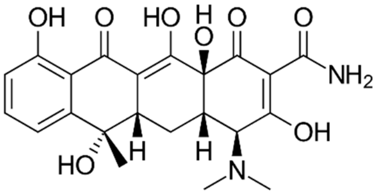
Sulfamethoxazole	Sul	723-46-6	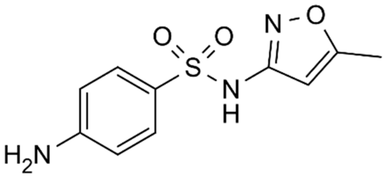
